# Comparative assessment of a restored and natural wetland using ^13^C-DNA SIP reveals a higher potential for methane production in the restored wetland

**DOI:** 10.1128/aem.02161-24

**Published:** 2025-02-06

**Authors:** Nora Hamovit, Taniya RoyChowdhury, Denise M. Akob, Xuesong Zhang, Gregory McCarty, Stephanie Yarwood

**Affiliations:** 1 Department of Biological Sciences, University of Maryland 1068 , College Park, Maryland, USA; 2 Department of Environmental Science and Technology, University of Maryland 542703 , College Park, Maryland, USA; 3 Geology, Energy and Minerals Science Center, U.S. Geological Survey, Reston, Virgnia, USA; 4 Agricultural Research Center, United States Department of Agriculture 57604 , Beltsville, Maryland, USA; Georgia Institute of Technology, Atlanta, Georgia, USA

**Keywords:** wetland soil microbial ecology, stable isotope probing, methanogenesis

## Abstract

Wetlands are the largest natural source of methane (CH_4_), a potent greenhouse gas produced by methanogens. Methanogenesis rates are controlled by environmental factors such as redox potential, temperature, and carbon and electron acceptor availability and are presumably dependent on the composition of the active methanogen community. We collected intact soil cores from a restored and natural freshwater depressional wetland on Maryland’s Delmarva Peninsula (USA) to assess the effects of wetland restoration and redox shifts on microbial processes. Intact soil cores were incubated under either saturated (anoxic) or unsaturated (oxic) conditions and amended with ^13^C-acetate for quantitative stable isotope probing (qSIP) of the 16S rRNA gene. Restored wetland cores supported a distinct community of methanogens compared to natural cores, and acetoclastic methanogens putatively identified in the genus *Methanosarcina* were among the most abundant taxa in restored anoxic and oxic cores. The active microbial communities in the restored wetland cores were also distinguished by the unique presence of facultatively anaerobic bacteria belonging to the orders *Firmicutes* and *Bacteroidetes*. In natural wetland incubations, methanogen populations were not among the most abundant taxa, and these communities were instead distinguished by the unique presence of aerobic bacteria in the phyla *Acidobacteria*, *Actinobacteria*, and class *Alphaproteobacteria*. Iron-reducing bacteria, in the genus *Geobacter*, were active across all redox conditions in both the restored and the natural cores, except the natural oxic–anoxic condition. These findings suggest an overall higher potential for methanogenesis in the restored wetland site compared to the natural wetland site, even when there is evidence of Fe reduction.

**IMPORTANCE:**

Methane (CH_4_) is a potent greenhouse gas with an atmospheric half-life of ~10 years. Wetlands are the largest natural emitters of CH_4_, but CH_4_ dynamics are difficult to constrain due to high spatial and temporal variability. In the past, wetlands were drained for agriculture. Now, restoration is an important strategy to increase these ecosystems’ potential for sequestering carbon. However, the consequences of wetland restoration on carbon biogeochemistry are under-evaluated, and a thorough assessment of the active microbial community as a driver of biogeochemical changes is needed. Particularly, the effects of seasonal flooding/drying cycles in geographically isolated wetlands might have implications for CH_4_ emissions in both natural and restored wetlands. Here, we found that active microbial communities in natural and restored wetlands responded differently to flooding and drying regimes, resulting in differences in CH_4_ production potentials. Restored wetlands had a higher potential for CH_4_ production compared to natural wetlands. Our results show that controls on CH_4_ production in a restored wetland are complex, and dynamics of active microbial communities are linked to seasonal dry–wet cycles.

## INTRODUCTION

Freshwater wetlands are the largest natural source of atmospheric methane (CH_4_), a potent greenhouse gas (1). Wetland CH_4_ emissions are a product of microbial CH_4_ production and oxidation in the soil and sediments (2). Emissions can be difficult to predict, however, as the underlying biogeochemical processes are influenced by several biotic and abiotic factors operating at various temporal and spatial scales (2, 3).

Wetland restoration can re-establish essential ecosystem services, such as carbon sequestration and water quality improvement, in the landscape (4). Past land use change and diverse restoration practices, however, can result in complex gradients of environmental and edaphic disturbances across sites with lasting impacts on the ecosystem function (5–8). The selection of soil substrates and restoration methods can further add to the complexity of constraining CH_4_ production and oxidation to account for greenhouse gas budget and carbon sequestration (4, 9). Evidence of this complexity includes observations of higher and more variable CH_4_ emissions from restored sites, relative to their natural counterparts (10–14).

**Fig 1:**
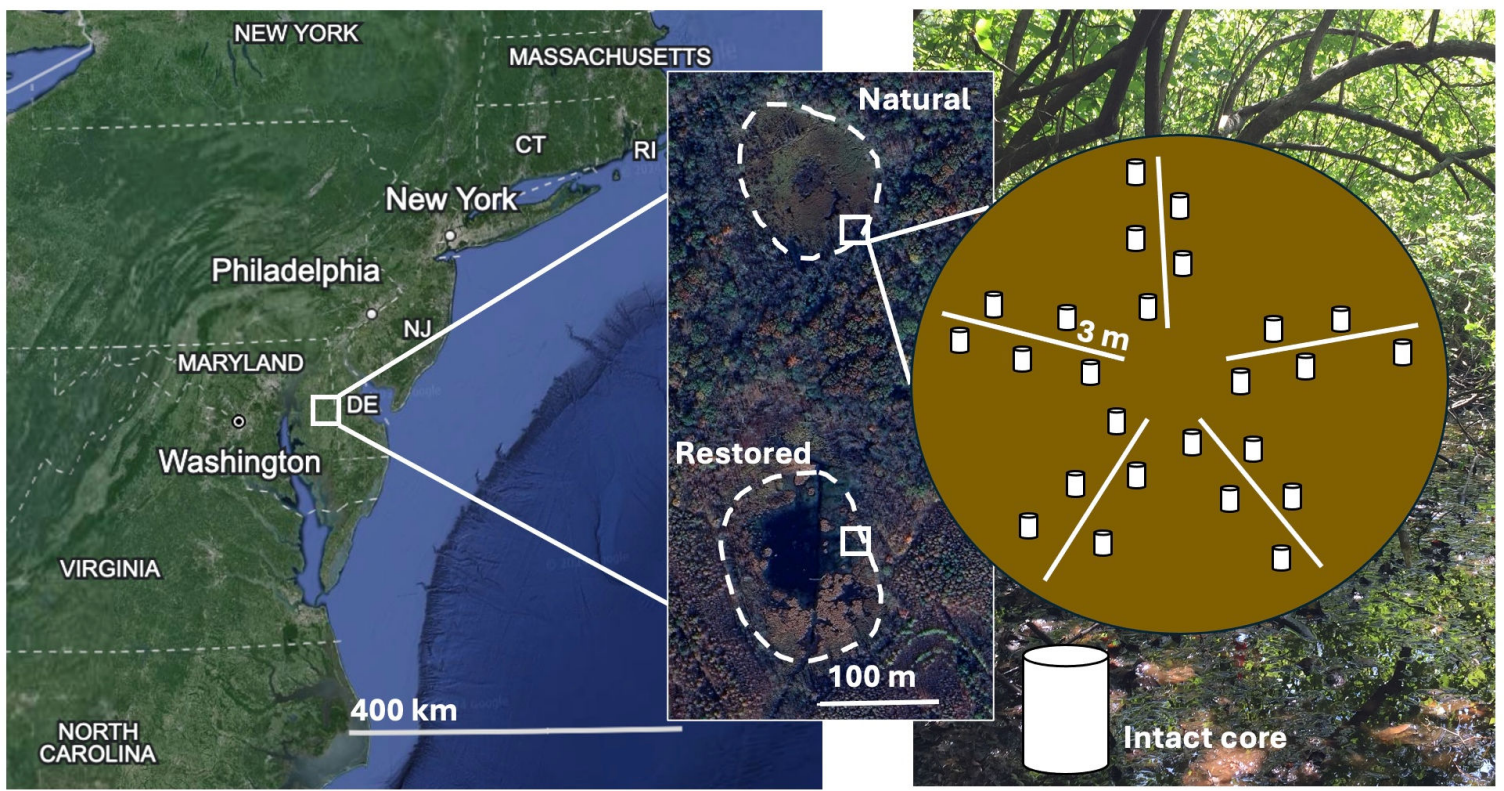
(Left) Map showing wetland sites located on Delmarva Peninsula in Maryland, USA. (Data from Google, Landsat/Copernicus, SIO, NOAA, U.S. Navy, NGA, GEBCO, LDEO-Columbia, NSF, 14 December 2015–1 January 2021.) (Center) Wetlands within sampling location at wet forest edge (white boxes) highlighted (data from Airbus, 9 November 2023). (Right) Example of transect design for soil core collection. Cores were collected every 0.5 m along five 3 m transects radiating from a randomly chosen location in the forested edge of the wetlands. Twenty-one and 27 cores were collected from the restored and natural sites, respectively.

Depressional freshwater wetlands, known as Delmarva Bays, occur across the coastal plain of Maryland’s Delmarva Peninsula (~3,000 sites) (15) (Fig. 1). These sites typically measure between 0.12 and 1.13 ha and are characterized by loamy soil textures, thin (<5 cm) organic horizons, and iron (Fe)-rich minerology (16–18). Delmarva Bays experience seasonal shifts in hydrology with a fall wet-up due to increases in groundwater level and rainfall (16, 19). They also experience a summer dry-down, creating drier edges and inundated centers (16, 19). The wet edge of Delmarva Bays may experience more frequent dry–wet cycles due to rainfall-induced flooding. Dry–wet cycles influence the redox state of Delmarva Bays, driving soils to oxic and anoxic conditions, respectively. The response of CH_4_ production to wet-up and dry-down events in seasonally flooded systems is variable and depends, in part, on the methanogenic Archaea present, creating further complexities in constraining CH_4_ emissions from seasonally flooded wetlands (20–23).

Delmarva Bays restored from agricultural land often exhibit soil homogenization and compaction, lower soil organic carbon content, increased herbaceous vegetation, and elevated pH compared to nearby natural wetlands (17, 24, 25). Disturbance from past agricultural land use, including soil homogenization, can change nutrient, carbon, and electron acceptor availability and composition (8, 26–29). Soil carbon chemistry and electron acceptor availability are key drivers of CH_4_ cycles. It is well established that methanogenesis is an energetically unfavorable process, and competition for C substrates with bacteria that utilize alternative terminal electron acceptors, such as Fe^3+^, can outcompete CH_4_ production (30–33). As such, altered microbial availability of Fe^3+^ in disturbed restored wetland soil could result in C substrates flowing disproportionately toward methanogenesis rather than Fe reduction (31, 32). Alternatively, fluctuations in hydrology, as well as corresponding cycles of Fe reduction and oxidation, at the edge of natural Delmarva Bays may support Fe^3+^ reducers’ utilization of competitive C substrates like acetate (31, 32). However, it is not known how the interplay of Fe–C substrates varies with the seasonal dry–wet cycles in Delmarva Bays and if such an interplay selects for specific microbial processes with consequences for the CH_4_ cycle. Therefore, in this study, we compare the shifts of the microbial community composition in the forested edges of natural and restored wetlands using simulated hydrology in the laboratory. Specifically, we focus our investigations on the active microbial communities as the primary contributing agents to CH_4_ biogeochemistry using laboratory-based experiments.

**Fig 2:**
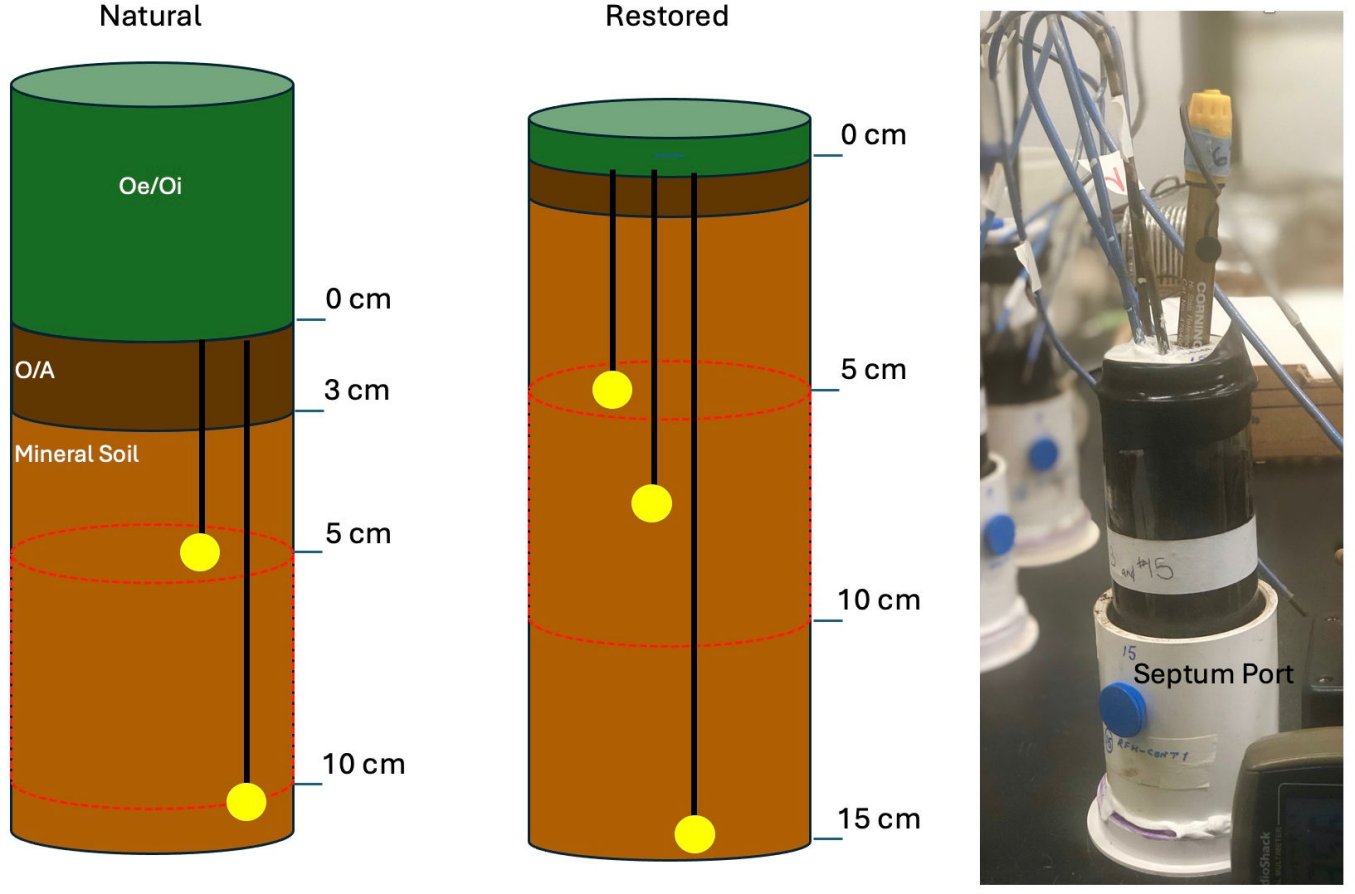
Schematic showing the differences between the intact soil cores obtained from the natural (left) and restored (right) wetlands. Natural wetland cores were shallower as a deeper organic layer (0–6 cm) on average was removed prior to incubation (green, Oe/Oi) compared to the restored wetland cores. The yellow circles represent the approximate locations of the redox probes: three probes were inserted in the restored wetland cores corresponding to 5.0, 7.5, and 15.0 cm depth below surface (bls). Approximate depths of redox probes for the natural wetland cores were 5 and 10 cm bls; there was no third depth due to the shallow nature of these cores. The equivalent depth range of comparison using DNA–SIP presented in this study is shown by red dotted lines. A photo of the cores placed in incubation design is shown.

We used a DNA-based quantitative stable isotope probing (qSIP) approach to determine shifts in community composition corresponding to shifts in redox conditions in a paired natural and restored Delmarva Bay (34). To do so, we collected intact soil cores using a custom-designed soil corer that could receive a glass tube for soil collection (Fig. 2; Fig. S1) from two Delmarva Bay wetlands, a natural site (referred to as “natural”) and a nearby site restored from agricultural land in 2004 (referred to as “restored”) (Fig. 1). We collected soil samples from the wetland’s forested edges (Fig. 1), where seasonal fluctuations in water levels and redox are most evident (19). In the laboratory, cores were incubated to simulate the following *in situ* conditions: (i) continuous saturation to mimic flooding (referred to as “anoxic”), (ii) summer draw-down to mimic soil drying (referred to as “oxic”), and (iii) draw-down followed by wet-up (referred to as “oxic–anoxic”). An isotopically labeled ^13^C-acetate solution was used as a substrate to examine the active microbial community composition through DNA-stable isotope probing (SIP) by comparison with ^12^C-acetate amended treatments. Acetate was chosen as acetotrophic methanogens can account for 60%–70% of all CH_4_ emitted in freshwater wetlands (35) and is a common substrate for multiple anaerobic pathways, including Fe reduction (32).

We hypothesized that (i) highly microbially available Fe^3+^ in the mineral soils of the natural wetlands would results in Fe-reducing bacteria outcompeting methanogens for acetate under flooded, anoxic conditions; in contrast, losses in microbially available Fe^3+^ in the restored wetland would allow methanogenesis to be more favorable under anoxic conditions; (ii) soil drying, simulating *in situ* seasonal draw-down, would select for aerobic microbial activity due to the resulting oxic conditions, and these shifts would be more pronounced in the natural wetland; (iii) re-wetting of dry soil to shift the conditions from oxic to anoxic in the natural wetlands would result in an increase in Fe-reducer populations compared to methanogens and increase evidence of Fe reduction.

## RESULTS

### Physiochemical characteristics of soil cores

#### Redox potentials during the laboratory incubations differed between soil cores from the natural and restored wetlands

**Fig 3:**
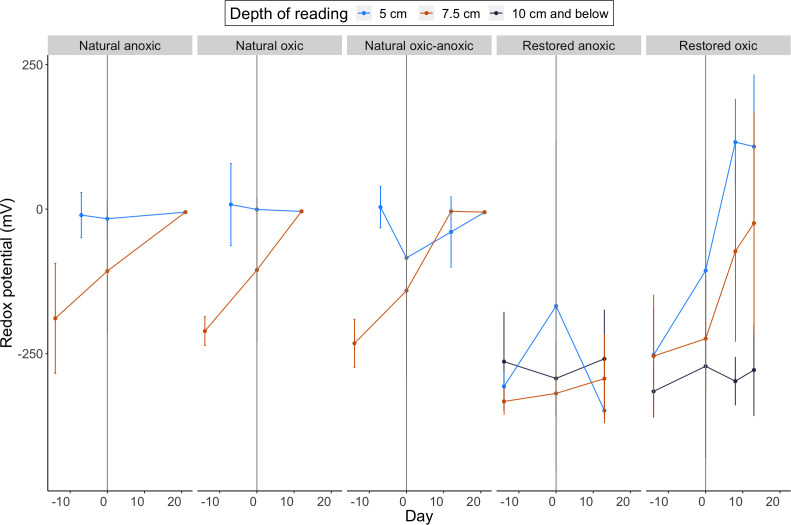
Change in redox potential (mV) during the soil core incubations. Data are presented as averages (±standard deviations) for replicate cores (*n* = 6) in each redox treatment group. Redox readings were collected at three depths within the core, as indicated by color. For all cores, the first redox measurement is reported from the pre-incubations (days −14 or −7), followed by a measurement made at the initiation (day 0) of the incubations. Vertical line at day 0 indicates the beginning of incubation. Midpoint redox measurements were made on day 8 in restored cores, followed by a final reading on day 13. The redox reading for all natural cores is reported for day 12. Natural anoxic and oxic–anoxic cores continued through day 21, when a final redox measurement was made. Data are facetted by wetland and redox conditions on the *x* axis.

Natural and restored cores had variability in the depth of soil layers present during incubation, as a deeper Oe/Oi layer in the natural soil cores collected during sampling was removed prior to incubation (Fig. 2) (Table 1; Fig. 3). As a result, the soil targeted for physiochemical analysis in all cores is meant to capture a uniform depth below the soil surface of the incubated cores (Fig. 2). Initial (day 0) redox readings at the targeted depth (Fig. 2) in the natural cores (−117.9 ± 127.1) were higher than those in the restored cores (−271.5 ± 70) (two-way analysis of variance [ANOVA], *P* < 0.001) (Table 1; Fig. 3). Natural wetland cores in all treatments increased over the incubation period of 12 (oxic) and 21 (oxic–anoxic and anoxic) days. As a result, final redox values at the target depth (7.5 cm below surface), assessed on the last day of incubations, did not differ between the natural redox treatments (Table 1; Fig. 3). Additionally, final redox readings in all natural treatments were higher than those in both the restored oxic and anoxic treatment cores at a depth of 7.5 cm below surface (Tukey’s test, *P* < 0.05) (Table 1). Final gravimetric water content (GWC) at the target depth did not differ with treatment in the natural cores despite being 7% and 6% lower in the natural oxic cores than in the natural anoxic and oxic–anoxic cores, respectively (Table 1).

**TABLE 1:** Mean (±standard deviation) redox, Fe^2+^, total Fe, pH, GWC, and DOC were assessed at depth used for DNA–SIP community analysis upon completion of incubation^*a*^

Type	Redox status	Conditions at depth used for DNA–SIP								Whole core	
		Redox (day 0) (mV)	Redox (midpoint) (mV)	Redox (end) (mV)	Fe^2+^ (µM/g dwt soil)	Total Fe (µM/g dwt soil)	pH	GWC (g/g)	DOC (mg/g dwt soil)	CH_4_ production rate (µmol/g dwt soil/day)	Final CH_4_ concentrations (µmol/g dwt soil)
Natural	Anoxic	−107.2 ± 106.5	NM	−5.2 ± 0.2	0.06 ± 0.08	0.33 ± 0. 3	4.6 ± 0.2	0.56 ± 0.21	0.37 ± 0.08	NM	BD
	Oxic	−105.4 ± 123.7	NM	−3.8 ± 0.2	15 ± 0. 09	0.43 ± 0. 2	4.9 ± 0.1	0.50 ± 0.12	0.22 ± 0.06	NM	BD
	Oxic–anoxic	−141.1 ± 151.2	−3.9 ± 0.4	−5.2 ± 0.3	05 ± 0.03	0.36 ± 0. 2	4.9 ± 0.5	0.57 ± 0.18	0.13 ± 0.04	NM	BD
Restored	Anoxic	−318.9 ± 38.7	NM	−293.4 ± 76.5	23 ± 0. 13	0.93 ± 0.8	5.4 ± 0.2	0.53 ± 0.07	0.19 ± 0.08	0.003 ± 0.006	0.14 ± 0.06
	Oxic	−224.2 ± 101.3	−72.9 ± 155.9	−24.2 ± 191.7	21 ± 0. 11	1.06 ± 0.4	5.3 ± 0.2	0.46 ± 0.13	0.23 ± 0.06	0.005 ± 0.004	0.08 ± 0.06

^*a*^ Concentration of CH_4_ was assessed in the headspace of each core (±standard error) on the final day of pre-incubations. Natural anoxic and oxic–anoxic incubations ran for 21 days, with a midpoint redox measurement on day 12 in the oxic–anoxic cores, after which they were re-wet. Natural oxic incubations ran for 12 days. All restored wetland incubations ran for 13 days, with a midpoint redox measurement made on day 8. Redox (mV) measurements were taken from the bottom (natural) and center (restored) of the depth used for DNA–SIP (see Materials and Methods). BD, below detection; DOC, dissolved organic carbon; dwt, dry weight; NM, not measured.

Redox values in restored oxic cores increased consistently at or above a 5 cm depth (Fig. 3), while redox values at the target depth (Fig. 2) varied between cores, resulting in a −24 mV mean redox potential at the end of the 13-day incubation (Fig. 3). Redox values in the anoxic restored cores remained consistently negative at the target depth and below (Table 1; Fig. 3). Readings remained consistent or showed a decrease at or above a 5 cm depth (Fig. 3). The final GWC was 7% lower in the restored oxic cores, on average, at the target depth interval than in the anoxic cores, but this again did not represent a significant decrease (Table 1).

#### End-of-incubation soil chemistry differed between the two wetlands

Soil pH measured at the end of incubations at the target depth were lower in the natural wetland cores compared to the restored wetland cores (two-way ANOVA, *P* < 0.0001) (Table 1). Soil pH also varied with a combined wetland type and redox treatment (*P* = 0.02), being lower under anoxic conditions in natural wetland cores (mean pH = 4.6) than in restored wetland cores (mean pH = 5.4) (Tukey’s test <0.05).

Concentrations of dissolved organic carbon (DOC) assessed at the end of incubations also varied with wetland type (two-way ANOVA, *P* = 0.002) and redox treatment (*P* < 0.001), as well as a combined wetland type and redox treatment (*P* < 0.001). Average DOC concentrations were higher in the natural wetland cores compared to the restored wetland cores (Tukey’s test, *P* = 0.002), but cores from both wetlands incubated under anoxic conditions showed evidence of higher DOC pools at the end of incubations compared to corresponding oxic cores (Tukey’s test, *P* < 0.01) (Table 1). Specifically, average DOCs measured at the end of incubation in the natural oxic cores were 38.5% and 43.3% lower than those in the anoxic and oxic–anoxic cores, respectively, while DOC in the restored oxic cores was 21.5% lower than that in the anoxic cores (Table 1).

Total dithionite–citrate (DC) extractable Fe measured at the end of incubations was lower in the natural wetland cores compared to the restored wetland cores (two-way ANOVA, *P* = 0.001) and did not vary with the redox treatment. Concentrations of aqueous Fe^2+^ (KCl extractable) were also higher in the restored cores compared to the natural cores (two-way ANOVA, *P* = 0.002) (Table 1) and again did not differ with the redox treatment. As a result, the ratio of total Fe to aqueous Fe^2+^ did not differ with wetland type or redox treatment, and aqueous Fe^2+^ and total Fe concentrations were positively correlated across the cores (*P* = 0.02, *R*^2^ = 0.17) (Fig. S5). At time zero of the incubations, CH_4_ was detected in the headspace of all restored cores but was below detection or quantification limits (0.0001% CH_4_) in all natural cores (Table 1).

### Active microbial communities in restored and natural wetland cores

#### Active Bacteria and Archaea were identified using qSIP analysis

At the end of the incubation period, DNA was extracted from the target depth for all restored and natural wetland cores receiving ^12^C-acetate and ^13^C-acetate and separated using density gradient ultracentrifugation (as described in the Materials and Methods) (Fig. S3). DNA from each sample was analyzed using qSIP, and amplicon sequence variants (ASVs) found to have a positive atom percent excess (APE) of ^13^C and a lower 95% confidence interval (CI) above zero APE were identified as the active portion of the community (34) (Table 2; Fig. S4). Abundance of taxa was corrected using 16S rRNA quantitative PCR (qPCR) data, allowing for the absolute abundance of active community members to be compared across wetland treatments (Table 2).

**TABLE 2:** The average number of active taxa (±SD), the average total absolute abundance (±standard error), and alpha diversity (SDI) (±SD) of the active communities in each wetland redox treatment (restored anoxic, restored oxic, and natural oxic, *n* = 3; natural anoxic and natural oxic–anoxic, *n* = 2)^*a*^

Type	Redox status	No. of active taxa	Total absolute abundance (gene copies/ng DNA)	Alpha diversity (SDI)
Natural	Anoxic	163 ± 3	785 ± 316	4.0 ± 0.1
	Oxic	70 ± 14	2,584 ± 1,802	3.3 ± 0.5
	Oxic–anoxic	54 ± 6	693 ± 640	3.2 ± 0.3
Restored	Anoxic	116 ± 33	2,014 ± 1,040	3.9 ± 0.4
	Oxic	127 ± 41	2,975 ± 1,688	3.7 ± 0.1

^*a*^ SD, standard deviation; SDI, Shannon diversity index.

#### The active Bacteria and Archaea community composition differed between the two wetlands

**Fig 4:**
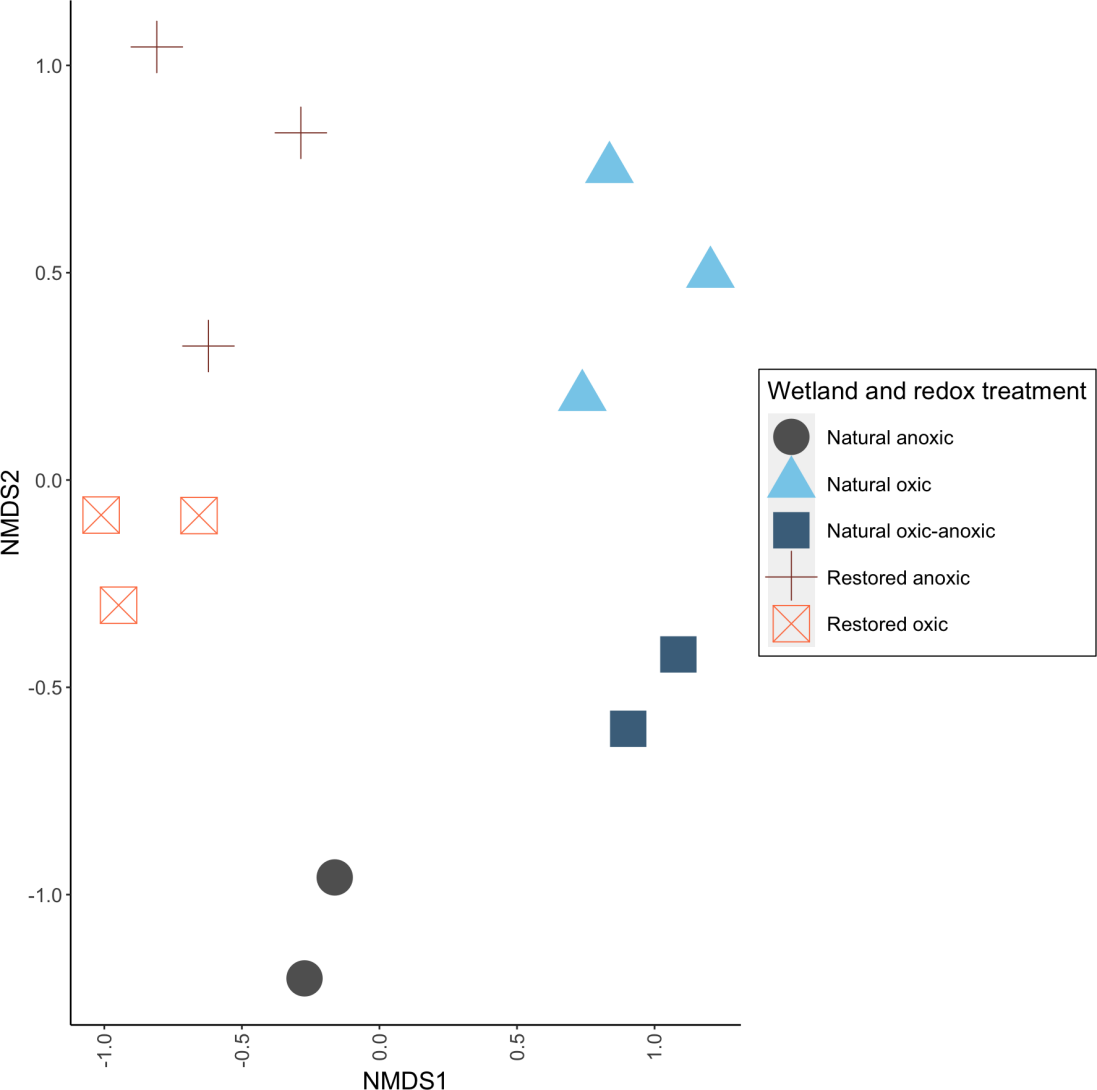
Non-metric multidimensional scaling (NMDS) of the active microbial communities in natural and restored wetland cores under different redox conditions. Points represent the Bray–Curtis distance between active communities in replicate cores. Community data reported as total absolute abundance of each active taxa in each ^13^C-treated core. *n* = 3 for the restored anoxic, restored oxic, and natural oxic cores, while *n* = 2 for the natural anoxic and natural oxic–anoxic cores.

Specifically, active community composition was distinct between restored and natural wetland cores (two-way permutational multivariate analysis of variance [PERMANOVA], *P* = 0.03, *R*^2^ = 0.14), although community was also structured by redox treatment (Fig. 4). The total absolute abundance of the active communities did not vary with wetland type or redox treatment but trended higher in the restored and natural oxic cores than in the corresponding anoxic treatments (Table 2). Differences in community diversity were more pronounced, and the average number of active taxa varied with redox treatment (*P* = 0.04), and a combined wetland type and treatment (*P* = 0.01). The number of active taxa, however, was only significantly higher in the natural anoxic active community compared to the natural oxic–anoxic (Tukey’s test, *P* = 0.03) and natural oxic (*P* = 0.05) active communities (Table 2). The calculated Shannon diversity index did not vary with wetland type or redox treatment but trended higher in the anoxic treatments (Table 2).

### Presence and abundance of phyla in the active communities

#### The average total absolute abundance of two phyla, *Actinobacteria* and *Chloroflexi*, differed between the wetland redox treatment active communities

**Fig 5:**
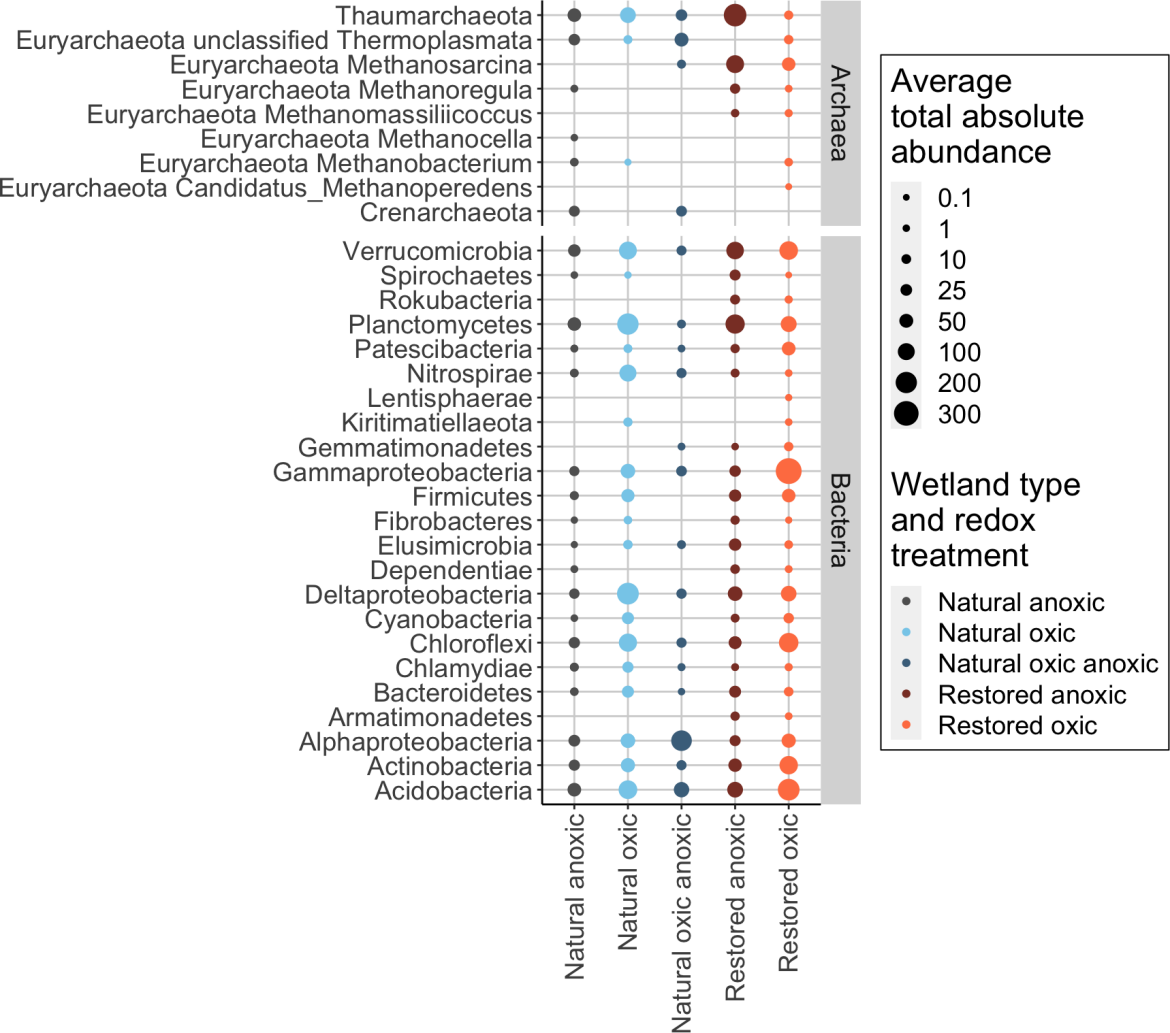
Average total absolute abundance of all active taxa grouped by phylum and by genera for methanogens in the phylum *Euryarchaeota*. Taxa are listed alphabetically by phylum and facetted by kingdom (Archaea and Bacteria). Size of the circle corresponds to average total absolute abundance in the wetland’s different redox treatment communities. Colors indicate wetland type and redox condition. Conditions are listed left to right: natural oxic, natural oxic–anoxic, natural anoxic, restored oxic, and restored anoxic.

Specifically, both *Actinobacteria* (Kruskal–Wallis chi-square = 11.4, df = 4, *P* value = 0.02) and *Chloroflexi* (chi-square = 9.8, df = 4, *P* value = 0.05) had an elevated total absolute abundance in the restored oxic active communities (Fig. 5). In addition, the phylum *Dependentiae* (Kruskal–Wallis chi-square = 7.8, df = 2, *P* value = 0.02), found only in the restored oxic, restored anoxic, and natural anoxic active communities, also varied with wetland type and redox treatment and was elevated in the restored anoxic communities (Fig. 5). The phylum *Rokubacteria* (Kruskal–Wallis chi-square = 4.4 , df = 1, *P* value = 0.04) was also elevated in the restored anoxic active communities compared to the restored oxic active communities (Fig. 5).

### Shared and unique active taxa across redox treatments

#### One hundred and twenty-one taxa were unique to restored core communities, and 32 of these taxa were found in both restored redox treatments

**Fig 6:**
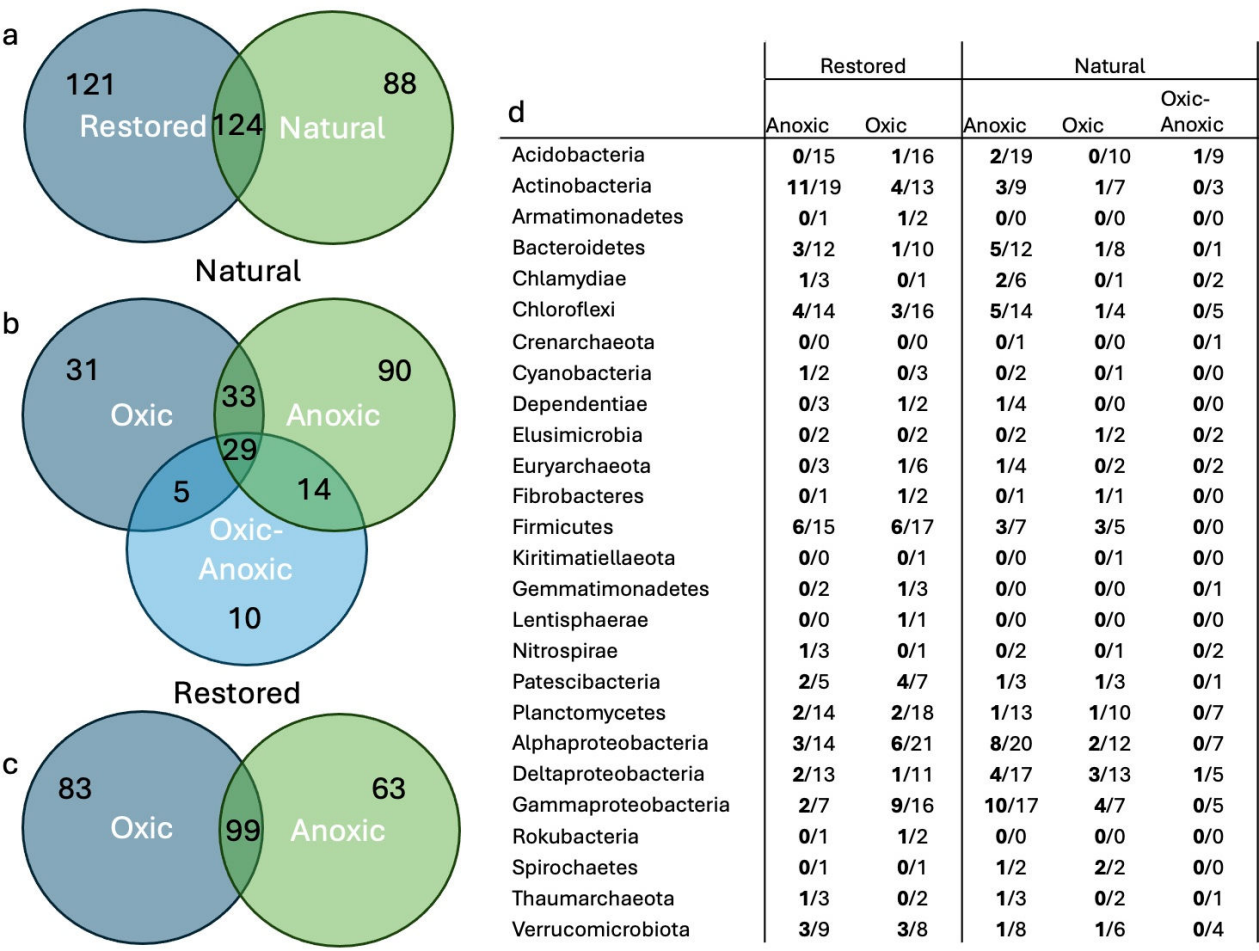
Venn diagrams showing the number of active taxa that co-occur or are independent in (a) active communities from the restored or natural wetland; (b) the natural oxic, anoxic, and oxic–anoxic treatments; and (c) the restored oxic and anoxic treatments. Numbers in the Venn diagrams report the number of taxa shared or unique to the groups compared. The accompanying table (d) lists the number of unique taxa over the total number of taxa (no. of unique taxa/no. of total taxa) in each wetland redox treatment group organized by phyla. The restored anoxic active communities had 42 unique taxa and 162 total taxa. The restored oxic active communities had 47 unique taxa and 182 total taxa. The natural anoxic active communities had 48 unique taxa and 166 total taxa. The natural oxic active communities had 22 unique taxa and 98 total taxa. The natural oxic–anoxic active communities had two unique taxa and 58 total taxa.

These taxa included methanogens putatively identified in the genus *Methanomassiliicoccus*, bacteria in the family *Methylomirabilaceae* associated with methanotrophy, members of the *Clostridiales* family *Ruminococcaceae*, members of the *Acidobacteria* class *Holophagae*, and *Chloroflexi* in the groups KD4-96 and JG30-KF-AS9 (Fig. 6; Table S4). Other methanogenic genera found in the restored anoxic and oxic active communities included *Methanosarcina*, which was among the most abundant genera in both restored redox treatments, and *Methanoregula* (Fig. 5). The genus *Methanosarcina* was also present in the natural oxic–anoxic active communities, while the genus *Methanoregula* was also found in the natural anoxic active communities (Fig. 5). The restored oxic active communities also included methanogens in the genus *Methanobacterium* (found also in the natural anoxic and oxic communities) and unclassified *Thermoplasmata* (found in all communities except for the restored anoxic communities) (Fig. 5; Table S4).

The restored anoxic active communities contained a total of 162 taxa present across 23 phyla, with the greatest number of unique taxa being found in the phyla *Actinobacteria* and *Firmicutes* (Fig. 6). Taxa unique to restored anoxic communities (*n* = 42) (Fig. S6) included members of the *Clostridiales* family *Ruminococcaceae*, as well as *Desulfovibrio* and *Syntrophorhabdus* in the *Deltaproteobacteria* class (Fig. 6; Table S4). The restored oxic communities contained a total of 182 taxa present across 25 phyla (Fig. 6). The greatest number of unique taxa was found in the class *Gammaproteobacteria* and included members of the family *Methylophilaceae*, known to contain aerobic methanotrophs. Additional genera unique to restored oxic active communities included the *Euryarchaeota* candidate genus “*Candidatus* Methanoperedens” and additional members of the *Clostridiales* family *Ruminococcaceae*, as well as members of the group *Clostridium sensu stricto 10* and *NC10* bacteria in the phylum *Rokubacteria* (Fig. 6; Table S4).

#### The natural wetland cores had 88 unique taxa identified as active, and while 29 taxa were found in all three natural redox treatments, no taxa were common to just these three treatments

Similarly, while the natural oxic–anoxic and anoxic active communities shared a total of 43 taxa, only 6 were unique to just these two groups, including genera in the phylum *Crenarchaeota* (Fig. 6; Table S4; Fig. S6). All other taxa were also found in the restored active communities (Fig. 6; Fig. S6). This was also seen for the natural anoxic and oxic active communities, which shared 62 taxa, of which only 9 were unique to these two treatments (Fig. 6; Fig. S6). These unique taxa included the genera *Desulfobacca*, *Desulfomonile*, and *Desulfovirga* in the class *Deltaproteobacteria*, as well as *Alphaproteobacteria* in the family *Acetobacteriaceae*, and *Clostridia* in the group *Clostridium sensu stricto 12* (Fig. 6; Table S4). Only members of the *Elusimicrobiota* group lineage IIa were unique to the natural oxic and natural oxic–anoxic active communities, although they shared 34 taxa (Fig. 6; Table S4).

The natural anoxic active communities contained a total of 166 taxa present across 21 phyla, with the greatest number of unique taxa being found in the phylum *Acidobacteria* and classes *Alphaproteobacteria*, *Deltaproteobacteria*, and *Gammaproteobacteria* (Fig. 6; Table S4). The taxa unique to the natural anoxic active communities (*n* = 48) (Fig. S6) included *Euryarchaeota* in the genus *Methanocella*, *Acidobacteria* in the family *Acidobacteriaceae* subgroup 1, *Methylobacterium* in the class *Alphaproteobacteria* , and members of the order *Clostridia* in the group *Clostridium sensu stricto 8*. The natural oxic active communities contained a total of 98 taxa present across 20 phyla, with the greatest number of unique taxa being found in *Alphaproteobacteria* and *Deltaproteobacteria* classes (Fig. 6; Table S4). Among taxa unique to the natural oxic active communities (*n* = 22) (Fig. S6) were members of the phylum *Verrucomicrobiota* in the family *Methylacidiphilaceae*, bacteria in the *Clostridia* family *Thermoanaerobacteraceae*, and the genus *Desulforhopalus* in the class *Deltaproteobacteria* (Fig. 6; Table S4). The natural oxic–anoxic active communities had a total of 58 taxa identified across 17 phyla, but only 2 were unique to this treatment, *Acidobacteria* in the family *Solibacteraceae* subgroup 3 and *Deltaproteobacteria* in the group P3OB-42 (Fig. 6; Table S4).

Under oxic conditions, the restored and natural wetland active communities had 59 common taxa, but only 4 taxa were unique to just these two communities (Table S4). These included *Actinobacteria* in the genus *Rothia* and members of the *Bacteroidetes* order *Chitinophagales*. Similarly, restored anoxic and natural anoxic cores shared 76 taxa with only 11 being unique to just these two communities. These included *Acidobacteria* in the family *Acidobacteriaceae* subgroup 1 and members of the phyla *Dependentiae* (Table S4). The natural oxic–anoxic and restored anoxic communities shared 37 active genera, with only 1 being unique to just these two redox conditions (*Chloroflexi* in the group TK10). Similarly, the natural oxic–anoxic and restored oxic communities shared 42 active taxa, with only 2 being unique to these two redox conditions, both in the phylum *Planctomycetes*. All active communities included taxa in the *Thaumarchaeota* group 1.1c, as well as members of the phylum *Planctomycetes* in classes *Planctomycetacia* and *Phycisphaerae*, and *Acidobacteria* in the class *Acidobacteriia* (Fig. 6; Table S4).

## DISCUSSION

### Active methanogenic Archaea and potential syntrophic activity

The incorporation of ^13^C-acetate into *Methanosarcina* under both redox conditions in the restored wetland cores suggested that a large portion of acetate was used for acetoclastic methanogenesis (Fig. 5). Incorporation of ^13^C-acetate into *Methanosarcina* was particularly pronounced in the restored anoxic core communities, with *Methanosarcina* as the third most abundant taxa (118.9 ± 174.6 gene copies per ng DNA) (Fig. 5). This high abundance is consistent with restored wetland cores producing CH_4_ during the pre-incubation under flooded conditions (Table 1) and suggests *Methanosarcina* were already active when ^13^C-acetate was added. Based on these observations, we infer that *Methanosarcina* are active under flooded conditions *in situ*.

A small number of *Methanosarcina* (6.2 ± 8.2) were also active in the natural oxic–anoxic communities (Fig. 5), suggesting that in the natural wetland, *Methanosarcina* may be less active (or inactive) under continuously flooded conditions but increase in activity upon re-wetting. This fits with observations of higher oxygen tolerance within the *Methanosarcina* genus (20), which may have allowed these organisms to respond quickly to high acetate availability during the re-wetting event. This may also explain why *Methanosarcina* remained active and abundant (45.1 ± 34.5) in restored oxic cores. While other methanogenic pathways may be active under continuously flooded conditions in the natural wetland, the absence of measurable CH_4_ from these cores during the flooded pre-incubation indicates that CH_4_ production is lower overall under flooded conditions in the natural wetland compared to the restored wetland. This could result in natural wetland sites having lower CH_4_ emissions during prolonged seasonal saturation (2).

Acetoclastic methanogens in the genera *Methanosaeta* were not found in any of the active soil core communities. Their absence may be a result of methanogens in this genus, having a lower affinity for acetate and longer doubling times than *Methanosarcina* (36). Sequences putatively matching hydrogenotrophic methanogens (i.e., *Methanobacterium*, *Methanoregula*, and *Methanocella*) were present in all cores, although at a consistently lower abundance than *Methanosarcina* (Fig. 5). Many hydrogenotrophic methanogens require organic substrates, such as acetate, for growth, but ^13^C labeling of their biomass is more commonly observed when exposed to ^13^C-CO_2_ (37). Production of ^13^C-CO_2_ in our incubations, resulting from microbial oxidation of ^13^C-acetate, is likely, given the length of the incubations. The potential for cross-feeding allows for detecting methanogenic pathways besides acetoclastic methanogens.

Syntrophic acetate-oxidizing bacteria (SAOB) convert acetate to CO_2_ for use by syntrophic partners (20, 38–41), and sequences putatively matching SAOB were found in the active communities of all wetland redox treatments. For instance, in restored anoxic active communities, the family *Ruminococcaceae* (order *Clostridiales*) was observed, which has been indicated as possessing SAOB (42, 43) (Table S4). In one study, syntrophic activity between *Ruminococcaceae* and *Methanosarcina* (performing hydrogenotrophic methanogenesis) was observed even when acetate was added (42), raising the possibility of syntrophic activity between SAOB and *Methanosarcina* under conditions favoring acetotrophic methanogenesis (42, 44). Alternatively, hydrogenotrophic methanogens, belonging to *Methanobacterium* and *Methanoregula*, were identified in the restored oxic and natural anoxic communities, alongside unclassified *Clostridiales* in *Clostridium sensu stricto* genera, which have also been proposed as potential SAOB (42, 45) (Fig. 5; Table S4). The potential importance of SAOB for converting acetate to CO_2_ for use by their syntrophic partners has challenged the perceived dominance of acetotrophic methanogenesis for acetate utilization (20, 38–41).

A small number of methylotrophic methanogens were identified in the restored cores in the genera *Methanomassiliicoccus*. Unidentified *Thermoplasmata*, which contains the genus *Methanomassiliicoccus*, however, were uniquely abundant in the natural oxic–anoxic communities (50.3 ± 67.7) and were the fourth most abundant taxa overall (Fig. 5). This could suggest a role in CH_4_ production for these Archaea within the natural wetland cores, particularly upon initial re-wetting. A lack of active Fe-reducing bacteria (i.e., *Geobacter*) and little evidence of Fe reduction (Table 1) in the natural oxic–anoxic cores further suggest that the production of CH_4_ outcompeted Fe reduction upon initial re-wetting in the natural cores (Table S4).

### Evidence of aerobic and anaerobic methanotrophy

Active aerobic methanotrophs were present across all redox conditions in both wetland core types, indicating that all incubations produced labeled ^13^C-CH_4_, via methanogenesis, and a portion of that CH_4_ was subsequently consumed. The consumption of CH_4_ produced during the incubations is most likely a result of long incubation times (2–3 weeks). Active methanotrophs in the *Alphaproteobacteria* genera were primarily found in the restored anoxic and/or natural anoxic cores (i.e., *Methylocystis* and *Methylobacterium*) (Table S4). Members of this class can be more tolerant to long-term flooding, due to an ability to form desiccation resistance structures (46–48), which could explain their presence in these soils that experience fluctuating hydrology *in situ*. Alternatively, active methanotrophs in the class *Gammaproteobacteria*, particularly in the family *Methylophilaceae,* were unique to the restored oxic communities, which could be a result of their ability to respond more quickly to CH_4_ availability post-disturbance (i.e., flooding or desiccation) (47) (Table S4). Aerobic methanotrophs in the *Verrucomicrobiota* class were unique to the natural oxic cores (Table S4). These methanotrophs are known to live in more acidic environments, which could explain their abundance in the natural cores, which had consistently lower soil pH^49^ (Table 1).

Only restored active communities had sequences putatively matching anaerobic methanotrophic archaea (ANME), again suggesting that these incubations produced labeled ^13^C-CH_4_, via methanogenesis, and a portion of that CH_4_ was subsequently consumed, even under anoxic conditions (Table S4). Specifically, Bacteria in the *Rokubacteria* NC10 family, *Methylomirabilis*, and Archaea in the genus “*Candidatus* Methanoperedens” are both known to perform nitrate-dependent anaerobic methane oxidation (49) and were present in the restored oxic and anoxic active communities (Fig. 5 and 6)

### Varying redox dynamics in the two wetland types informed the active communities

The restored cores exhibited slower drying (e.g., shift to oxic conditions) and lower redox values compared to natural oxic cores (Table 1). Two restored oxic cores, one receiving ^13^C-acetate and one receiving ^12^C-acetate, never achieved a redox reading above −180 mV at the depth DNA–SIP was performed (Fig. 4). Slow drying of restored cores may be due to increased soil compaction that has been observed in restored Delmarva Bays due to both agricultural land use and heavy machinery utilization during the restoration process (17). Soil compaction by heavy machinery has been linked to elevated CH_4_ emissions in forest soils, where decreased pore space slowed water movement through the soil and increased anoxia (50, 51).

Slow establishment of oxic conditions in the restored cores likely created more favorable conditions for obligate and facultative anaerobes throughout the incubation period, including the dominant methanogenic genus *Methanosarcina*. This is evidenced by the unique presence of obligate and facultative anaerobes, as well as microaerophilic acetate-utilizing bacteria, in the restored anoxic and oxic active communities. These community members include bacteria in the phyla *Firmicutes* (orders *Bacillales* and *Clostridiales*) and *Bacteroidetes* (orders *Bacteroidales* and *Kryptoniales* and group OPB56) (Fig. 6; Table S4). Members of these phyla have been shown to increase in anaerobic digesters exposed to microaerophilic conditions and may protect methanogens by consuming excess oxygen (45, 52, 53). Members of these phyla may also facilitate methanogenesis through production of substrates such as CO_2_ (45, 53), thereby contributing to methanogenesis via cross-feeding.

Alternatively, the rapid establishment of oxic conditions in all natural wetland cores likely led to more aerobic metabolism. As evidence, natural active communities uniquely shared several taxa in the phyla *Acidobacteria* (i.e., family *Solibacteraceae* and genera *Edaphobacter* and *Acidipila*) and *Actinobacteria* (i.e., genera *Skermania*, *Galbitalea*, and *Herbidospora*), which commonly contain aerobic bacteria (Fig. 6; Table S4). Additionally, *Alphaproteobacteria* in the family *Acetobacteraceae* were unique to the natural wetland core active communities and contain acetic acid bacteria, which are aerobic, acidophilic, and use sugars to produce acetic acid (54). The presence of these bacteria in only the natural wetland core active communities may also be partially explained by the lower pH of the natural cores (Table 1). Soil pH is a strong influence of microbial community composition, with a study comparing the microbial communities of North Carolina coastal plain natural and restored wetlands and agricultural sites finding pH to be the primary predictor of community composition (26).

### Evidence of fe reduction and active Fe-reducing bacteria

Active Fe reduction in our cores is evidenced by the presence of Fe^2+^ (Table 1) and the comparative ratio of total Fe to aqueous Fe^2+^ between the restored and natural cores (Fig. S5). The active communities (all but natural oxic–anoxic) also possess possible Fe-reducing bacteria, specifically those putatively matching the genus *Geobacter*, which could further suggest active Fe reduction. Together these data suggest Fe reduction occurred in cores even where active methanogenesis was evidenced, contradicting our prediction that Fe reduction would be limited in the restored cores, relative to the natural cores. Instead, our results suggest Fe reduction and methanogenesis co-occurred in the restored wetland cores. The high availability of acetate could account for the reduction in competition, meaning this dynamic may not be reflected *in situ*. Our results do suggest, however, that Fe reduction may still limit methanogenesis in the natural wetland *in situ*, as the abundance of active methanogens remained lower than potential Fe reducers (i.e., *Geobacter*), and natural cores showed more evidence of Fe reduction than methanogenesis (Table 1).

Further contradicting our predictions, both the restored and natural oxic treatments showed evidence of increased active Fe reduction (i.e., increased Fe^2+^ and/or increased *Geobacter* abundance) (Fig S5; Table S4) relative to their respective anoxic treatments. This indicates that increases in redox potential did not limit Fe reduction and instead may have allowed bacteria associated with Fe reduction to more competitively use acetate. An absence of active *Geobacter* and an increased abundance of active *Methanosarcina* in the natural oxic–anoxic communities may also contradict our prediction that Fe reduction would increase rapidly upon initial re-wetting in the natural wetland’s cores. Instead, these results suggest that the potential for CH_4_ production *in situ* may be highest upon initial re-wetting in natural wetlands.

### Conclusions

The structure of the active microbial community was distinct in the cores of a wetland 15 years post-restoration compared to those of a nearby natural reference. Restored active communities had higher methanogen abundance and unique populations of obligate and facultative anaerobic Bacteria and Archaea. A high abundance of *Methanosarcina* in the restored anoxic and oxic cores specifically suggested high levels of acetotrophic methanogenesis that continued during soil drying. This could inform high CH_4_ emissions from the restored wetland (55). While restored cores were slower to establish oxic conditions than natural cores, a shift in the active community members present in both restored and natural oxic cores compared to anoxic cores suggests that seasonal drying in these sites likely impacts the active community, although in disparate ways. Understanding how dry–wet events shift the active community and the potential for CH_4_ production in these sites has important implications for similar seasonally inundated wetlands. Additionally, our work demonstrates that future research is needed to optimize restoration in such a way to limit methanogen communities, particularly in soils that experience hydrologic fluctuations. This could help inform restoration policy, as current policy targets do not include carbon emissions and sequestration and instead focus on other ecosystem services (17, 56).

## MATERIALS AND METHODS

### Site description and soil collection

Soil was collected from a restored and natural forested wetland near Goldsboro, MD, USA (39°03′27″°N, 75°45′12″’°W). The wetlands have similar plant communities and vegetation cover, including trees and woody bushes. The typical hydrology in natural Delmarva Bays is described in Maietta et al. (16), and the hydrology of the sites is described in McDonough et al. (19). Wetland restoration from agricultural land occurred in 2004. The restored site was sampled in October 2018, and the natural wetland was sampled in August 2019. Water level depth in these sites is typically highest in the fall and winter (late October–March), drawing down during the growing season (16). The surface soils were inundated at the time of sampling in both the restored and natural wetlands.

Intact soil cores were collected at the wet forested edge of both wetland sites (Fig. 1), which experiences annual variability in water table depth, leading to fluctuation in soil redox conditions (16, 19). Cores were collected along five 3 m transects radiating from a randomly chosen undisturbed location in proximity to the edge of the wetlands (Fig. 1). A soil core was collected every 0.5 m along the transect, avoiding large trees/roots, to capture spatial heterogeneity (Fig. 1). Intact cores were collected using pre-sterilized glass tubes (15 × 2.54 cm diameter) and a custom-designed soil corer (Fig. S1).

Prior to coring, the top layer of surface organic matter (Oi/Oe layers) which contained undecomposed organic material (including leaves and roots) was removed to target the mineral soil layers for experiments (Fig. 2). The natural wetland cores had larger Oi/Oe layers resulting in an average intact core height of ~11 cm (corresponding to 5–11 cm below surface [bls] *in situ*), while 15 cm of core was recovered for the restored wetland (corresponding to 0–15 cm bls *in situ*). In between each sample collection, the soil corer was cleaned with 70% isopropanol, and each core was wrapped in pre-sterilized aluminum foil after extrusion. Cores were stored upright in a cooler at 4°C for transport to the lab. A total of 21 and 27 cores were collected from the restored and natural sites, respectively (Table 2).

### Soil core incubations

Cores were stored at 4°C until use for the soil incubations; incubations were run within 1 month of soil collection. Incubation experiments focused on the 5–10 cm depth of the soil core for both the restored and natural wetlands (Fig. 2). Intact soil cores were placed on custom-built polyvinyl chloride (PVC) stands and capped to establish an airtight headspace (Fig. S1). Caps were installed with septa for headspace gas sampling, and three platinum (Pt) redox probes were placed to monitor redox in the cores. Probes were placed at 5.0, 7.5, and 15.0 cm bls soil depth in the restored cores and at 5.0 and 10.0 cm bls soil depth in the natural cores (Fig. 2). The lowest depth for the probes corresponded to the bottom of the core for each site.

The PVC stands served as reservoirs for water and/or substrate (acetate) addition as described below. Pt redox probes were constructed and screened according to Mueller et al. (57). A reference calomel electrode was also installed in each core, and data were collected manually from all cores throughout the incubations to establish redox status (50) (Fig. 3). Cores were pre-incubated at their *in situ* GWC (~50% water weight [wt/wt]) in an anaerobic (N_2_) glove bag (Thermo Scientific Oxoid AnaeroGen 2.5L Sachet, Cole–Parmer Glove bag #0440860) at room temperature to acclimate the samples and establish a baseline methanogenic redox condition (Fig. 3).

During pre-incubation, redox measurements were taken weekly from the cores’ deepest redox probe (data not shown). Once cores had a consistent redox of −300 mV at this depth (around day 40), experimental manipulations began. Headspace CH_4_ measurements (0.5 mL subsamples) were made using gas chromatography with a flame ionization detector and methanizer (SRI 8610C; SRI Instruments, Torrance, CA, USA). Measurements were made during the pre-incubations beginning 14 days before incubation day 0 (day −14), followed by a measurement on day −8 and incubation day 0. All CH_4_ data were corrected for temperature and headspace volume and are reported here as micromole CH_4_ per gram dry weight (dwt) soil per day. Rates of CH_4_ production were calculated for each core using R studio (v.4.6.1, 2022.07.2) package *stats* function *lm* (v.4.2.2) and then averaged for each redox condition in both wetland types (Table S1).

Three cores from both wetlands were destructively sampled as time zero (*T*_0_) and stored at −20°C until further processing. The other restored wetland cores were separated into the following treatments: six cores were amended with 20 mM natural abundance acetate (CH_3_^12^COOH), 20 mM 99.99 atom % ^13^C-acetate (CH_3_^13^COOH), or distilled (DI) H_2_O. Similarly, natural wetland cores were also separated and amended with the same three solutions. All solutions were autoclaved and degassed immediately before addition. Half of each solution was added at the top of the cores using a sterile glass pipette, and the other half was added from the bottom via a septum at the bottom of the core stand (Fig. 2) to ensure submerged conditions and optimize exposure of the soil to the added substrates. Cores were incubated according to the experiment treatment described below. Total incubation times ranged from 12 to 21 days, depending on the time needed for the soil systems to arrive at the target redox potentials reflective of anoxic, suboxic, or oxic conditions.

#### Restored oxic condition

After substrate additions were made, three restored cores from each of the three solution addition types (*n*_13C-acetate_ = 3, *n*_12C-acetate_ = 3, *n*_DIH20_ = 3) were removed from the anaerobic glove bag. These cores were left under ambient aerobic conditions to establish oxic conditions in the top 5 cm of the cores over time. This would simulate the *in situ* summer draw-down in water levels (Fig. S2). Redox potentials were monitored regularly at the three core depths (Fig. 3). It took up to 13 days for the redox potential to reach values for oxic conditions (average redox was recorded at 93.5 ± 100.4 mV) in the top 5 cm of these cores. Cores were frozen for subsequent deconstruction as the restored-oxic condition (*n*_13C-acetate_ = 3, *n*_12C-acetate_ = 3, *n*_DIH20_ = 3).

#### Natural oxic condition

After substrate additions, six natural cores from each of the three addition types (*n*_13C-acetate_ = 6, *n*_12C-acetate_ = 6, *n*_DIH20_ = 6) were removed from the glove box and left to dry under ambient conditions to establish oxic treatment conditions in the top 5 cm of the cores over time. This would simulate the *in situ* summer draw-down in water levels (Fig. S2). Redox potentials were monitored regularly at the three depths (Fig. 3). It took up to 12 days for the redox potential to reach values for oxygenated conditions (average redox was recorded at −4.0 ± 0.2 mV) in the top 5 cm of these cores. Nine of these cores were frozen for subsequent destructive sampling for the oxic condition (*n*_13C-acetate_ = 3, *n*_12C-acetate_ = 3, *n*_DIH20_ = 3), while the remaining nine were used for the oxic–anoxic treatment.

#### Natural oxic–anoxic condition

Once oxic conditions were established in the six remaining oxic cores; i.e., after 12 days, they were re-wetted using the respective substrate solutions to simulate to *in situ* wet-up in early fall (Fig. S2). It took an additional 9 days for these cores to become anoxic (Fig. 3). At this point, they were frozen at −20°C until further processing. Only natural soil cores could be used for the oxic–anoxic condition.

#### Continuous anoxic condition

A continuous anoxic incubation was maintained in parallel to the oxic (restored and natural) and oxic–anoxic manipulations (for the natural only) inside the anaerobic glove box. These cores received the respective substrate solutions/water to maintain constant weight and were monitored for redox potential (Fig. 3). Restored anoxic cores were incubated for a total of 13 days to be consistent with the endpoint for the oxic incubations described above (Fig. 3). We did not have enough cores from the restored wetland that could be used for the oxic–anoxic manipulation. Natural anoxic cores were incubated for a total of 21 days for consistency with the total length of time required for the oxic–anoxic treatment’s incubation time. At the end of the incubation, all cores were frozen at −20°C until further processing.

### Core destructive sampling

Restored cores were destructively sampled by dividing them into three depths: 0–5 cm, 5–10 cm, and 10–15 cm. Natural wetland cores were ~11 cm in total depth and were divided into 0–5 cm and 5–11 cm (Fig. 2). To accurately compare the incubations from the restored and natural wetlands, only a single depth interval representing mineral soil was used for DNA–SIP analysis: these depth intervals corresponded to the 5–10 cm section from the restored wetland cores and the 5–11 cm section from the natural wetland cores. Redox (mV) conditions at the depth used for DNA–SIP analysis were monitored at a 10 cm depth in the natural cores and a 7.5 cm depth in the restored cores. SIP analysis was conducted only on these equivalent depths to best compare the active soil microbial community in the mineral soils of the restored and natural wetlands.

### Physicochemical analyses

The remaining soil representing the depth used for DNA–SIP from each core was homogenized, sieved (<2 mm), and stored (−40°C) wet for analysis. Soil GWC was assessed by drying 5 g of wet soil to a constant weight at 105°C. Soil pH was measured in a 1:5 wt:vol soil to DI water slurry and in a 1:5 soil to 0.1 M KCl slurry (2 g soil, 10 mL extractant). Soil and extractant were mixed by hand, allowed to settle, and measured using a pH meter (Accumet basic AB15, Devens, MA, USA) with reference points at pH 4, 7, and 10.

Total DC extractable Fe (measurement of both Fe^2+^ and Fe^3+^) was assessed on aliquots of oven-dried (105°C), ground, and sieved soil. Briefly, soil, sodium dithionite (Na_2_S_2_O_4_), and 0.5 M sodium citrate (Na_3_C_6_H_5_O_7_) solution were combined in a 1:1:60 vol:vol:vol ratio, respectively (1.25 mL soil, 1.25 mL sodium dithionite, and 75 mL sodium citrate) (58). The extractions were then shaken for 24 h at 200 rpm, followed by a 5 min centrifugation at 1,000 rpm. The supernatant was then filtered (Whatman #1 filter paper), and one drop of 16 M nitric acid was added to ensure stability during storage. Samples were diluted in a 1:201 vol:vol ratio with ultrapure water and analyzed in duplicate on an atomic absorption analyzer (PinAAcle 500, PerkinElmer Shelton, CT, USA; MDL ~ 0.02 mg/L). The concentration of total DC extractable Fe (Fe^2+^ and Fe^3+^) was back calculated to microgram per gram dwt soil using the soil GWC.

The mineral soils from each core were also subsampled for free aqueous Fe^2+^ analysis in an anaerobic (N_2_) glove bag (Thermo Scientific Oxoid AnaeroGenTM 2.5L Sachet, Cole–Parmer Glove bag #0440860). The Fe^2+^ was extracted in a 1:5 soil-to-2 M KCl ratio (59). The use of KCl was aimed at capturing aqueous Fe^2+^ that had likely been reduced during the incubation period. Soil slurries were shaken for 1 h at 60 rpm on a rotating shaker (Daigger Fine PCR series #EF4898C, Buffalo Grove, IL, USA). The slurry was filtered through a 0.2 µm nylon syringe filter and analyzed for free, aqueous Fe^2+^ using the 1,10-phenanthroline-based Hach FerroVer method 8146 (Hach Co., Loveland, CO USA). Samples were measured on a Hach spectrometer (Hach DR/4000) at 510 nm along with standards ranging from 0.02 to 3.0 mg L^−1^.

Soil DOC was extracted from 2 g of soil aliquoted from thawed incubation cores. Extractions were performed in a 1:5 w:v ratio of soil to 0.5 M K_2_SO_4_ (60, 61). Soil extractions were rotated at 30 rpm for 1 h on a rotating shaker (Daigger Fine PCR series #EF4898C, Buffalo Grove, IL, USA). Samples were filtered (Whatman 42 paper) and analyzed using a Shimadzu total organic carbon analyzer (TOC-LCPH/N, Shimadzu Cooperation, Japan) (62) with a standard curve ranging between 0.5 and 50 mg L^−1^. The concentration of DOC was back calculated to µg g^−1^ dwt soil using the soil GWC.

### DNA extraction and stable isotope probing

Soil DNA extractions were performed according to the QIAGEN DNeasy PowerSoil DNA extraction kit protocol (QIAGEN, Hilden, Germany). The DNA extracts were concentrated and precipitated using a Zymo Research DNA Clean & Concentrator kit and protocol (Zymo Research, Irvine, CA, USA). Soil DNA extracts were separated using cesium chloride (CsCl) density gradient centrifugation according to methods adapted from Neufeld et al. (63), Buckley et al. (64), and Beulig et al. (65). The CsCl gradients were formed by filling 5 mL polyallomer ultracentrifugation tubes (Thermo Fisher Scientific, Waltham, MA, USA) with a known density CsCl (7.163 M) solution and 1 µg DNA in gradient buffer (0.1 M Tris, 0.1 M KCl, and 1 mM EDTA) to obtain a homogenesis density of 1.725 g/mL. The tubes were sealed, balanced, and loaded into a S110-AT fixed angle rotor (Thermo Fisher Scientific). Density separation of DNA was achieved by centrifugation at 164,000 × *g*_max_ for 56 h in a Sorvall MTX 150 micro-ultracentrifuge (Thermo Fisher Scientific). These centrifugation parameters were confirmed to create a density gradient sufficient to separate pure ^12^C-DNA (unlabeled) and pure^13^C-DNA (labeled at 99 atom %) with average buoyant densities of 1.7 and 1.75 g/mL (63, 64).

Immediately after centrifugation, 12 fractions of 400 µL per tube were obtained using a syringe pump (Dual-NE-1000x; New Era Pump System, Farmingdale, NY, USA). The pump was run at a rate of 400 µL/min to dispense sterile DI water into the tops of sealed tubes and displace the fractions downward where they were collected into separate sterile 2 mL tubes. The density of each fraction was measured by refractive index using an AR200 digital refractometer (Reichert Technologies, Munich, Germany) modified for 5 µL of solution according to Buckley et al. (64). Refractive index was corrected for temperature (°C) and density of CsCl and gradient buffer addition (63).

DNA from each fraction was purified by polyethylene glycol (PEG) precipitation as described by Neufeld et al. (63). In brief, 1 µL of glycogen (Sigma cat. no. 10901393001) and 2 volumes of PEG solution (30% polyethylene glycol 6000, 1.6 M NaCl) were added to each fraction sample. The samples were inverted to mix, precipitated at room temperature for 2 h, and centrifuged at 16,000 × *g* for 30 min at 18°C. The pellet was washed in reagent grade, 70% ethanol and centrifuged again at 16,000 × *g* for 15 min at 18°C. The pellet was dried and re-suspended in 20 µL 1 M Tris–HCl (pH 8.5). Following precipitation, the DNA was quantified using high-sensitivity fluorometric quantification (Qubit [v.2.0] fluorometer; Invitrogen, Waltham, MA, USA). Average DNA recovery rate was 62.51% ± 18.85% for the restored wetland and 94.92% ± 14.29% for the natural wetland samples.

### 16S rRNA gene sequencing and qPCR

DNA recovered from CsCl gradients was prepared for downstream analysis by combining the 12 fractions for each technical replicate into three density (g/µL) groups: light (1.689–1.65 g/µL), intermediate (1.749–1.69 g/µL), and heavy (1.8–1.75 g/µL). DNA was concentrated using a Zymo DNA Clean & Concentrator-25 kit (cat. no. D4033; Zymo Research, Orange, CA, USA) (Fig. S2). Fraction groups were then prepared for sequencing on an Illumina MiSeq platform using 16S rRNA gene primers targeting the V3 region. The primers used were the Caporasso primer set M2347 (66): 515F (5′-GTGCCACGCMGCCGCGGTAA-3′) and 806R (5′-GGACTACHVGGGTWTCTAAT-3′). The PCR was performed using 7 µL of 1 mM of the forward/reverse primers with Illumina adapters (66), 17.5 µL of Phusion flash master mix (Thermo Fisher cat. no. F548S), and 3.5 µL of DNA. Two PCR clean-up steps were performed using AMPure XP beads (Beckman Coulter cat. no. A63881). Indexing was performed using Nextera XT 96 index kit (Illumina cat. no. 15052165). DNA was pooled and the amplicon size of the library was assessed through high-sensitivity fluorometric quantification (Qubit [v.2.0] fluorometer, Invitrogen). Final libraries were run on an Illumina MiSeq using a 600-cycle (v.3) cartridge at the University of Maryland Institute for Genome Sciences.

qPCR was performed using the same set of 16S rRNA gene primers (515F/806R) without Illumina adapters (66). The qPCR recipe for each sample well included 2 µL of DNA diluted to 1.25 ng/µL, 1 µL of 10 mM forward and reverse primers, 6 µL of sterilized MQ H_2_O, and 10 µL of KiCqStart SYBR Green qPCR ReadyMix with Rox (Sigma KCQ202-5000RXN LNQ66181567). Triplicate qPCR reactions for each sample were run on a StepOne Real-Time PCR System (Applied Biosystems, Waltham, MA, USA) according to the following thermocycling conditions: 95°C for 1 min, followed by 40 cycles of 95°C for 5 s, 55°C for 15 s, and 72°C for 10 s, followed by a final extension at 72°C for 10 min. A standard curve was constructed by serially diluting plasmid DNA containing the cloned 16S rRNA gene from *Escherichia coli*. A 10-fold dilution series was made, consisting of standards that ranged from 10^0^ to 10^−6^ gene copy numbers per microliter of solution. A second spiked standard curve with microcosm and plasmid DNA was used to account for sample-specific inhibition according to Hargreaves et al. (67). Standard curves had *R*^2^ values of >0.99 and efficiency values between 93% and 100% for the normal and spiked standards.

### Sequence analysis

Illumina sequencing outputs were processed using the dada2 package (v.1.26.0) (68). Sequences were filtered for quality, and adaptor and primer base pair were removed. Following this, error rates were calculated, allowing pair-end reads to be merged. The dada2 package was then used to remove chimeras and make taxonomic assignments in reference to the SILVA database (v.132, arb-silva.de). The resulting ASV table was analyzed using the phyloseq (v.1.38) (69), vegan (v.2.6–2) (70), and qSIP (v.0.1.0, https://github.com/bramstone/qsip) (34) R packages.

Rarefaction was not performed, but samples with outlier sequence depths were removed from analysis so that all samples had sequence depths within two standard deviations of the mean sequencing depth (93,838). At this time, four samples were removed from analysis. Rare and low abundance ASVs that did not appear at least three times in 2% of the samples were removed from analysis due to low confidence in accurate identification. The remaining ASV table, containing 8,551 ASVs, was transformed to relative abundance and normalized using 16S rRNA gene qPCR data following a QSEQ approach (71, 72). The ASV absolute abundance is thus reported as the number of gene copies per nanogram DNA in the combined fraction groups (heavy, medium, or light). During normalization, an additional 11 samples were removed from analysis due to insufficient qPCR data. Individual sample sequencing information can be found in the supplemental material (Table S2).

Normalized ASV tables from each density group (heavy, medium, and light) from each ^13^C and ^12^C-acetate replicate core in each redox condition from both wetlands were then fed into the qSIP pipeline (*qsip* R package [v.1.4.1]), which closely follows the steps laid out in Hungate et al. (34). The number of replicates for each density group within each acetate addition type, as well as each wetland redox condition group, can be found in the supplemental material (Table S3).

During qSIP, a taxon (ASV)-specific weighted average density (WAD) was calculated for each taxon in both the ^13^C-acetate- and ^12^C-acetate-treated replicate cores at the target depth in each wetland redox treatment group. At this time, an average overall WAD for the ^13^C-acetate- and ^12^C-acetate-treated replicates in each redox treatment was also calculated. All WADs were calculated in reference to the average density (temperature-corrected refractive index) of the fraction groups (heavy, medium, and light), and all WADs were bootstrapped (1,000 draws), giving an estimate of both the average WAD and the 95% confidence interval (34).

The difference in average WADs for each taxon was then calculated by subtracting the bootstrapped average WAD across control ^12^C-acetate replicates from the bootstrapped average WAD across^13^C-acetate replicates, as described in Hungate et al. (equation 4) (34). The calculated differences in WAD were also bootstrapped (1,000 draws), giving an estimate of both the average difference in WAD for each taxon and a 95% CI for each taxon. Taxon-specific GC content and molecular weight (g/mol) were then estimated based on the weighted average density in the ^12^C-acetate-treated cores using the linear relationship between GC content and buoyant density described in Hungate et al. (equation 5) (34). To estimate the APE of ^13^C for each taxon, we first calculated the molecular weight of the taxon in the ^13^C treatment using the proportional increase in density of the taxon relative to the density in the unlabeled ^12^C treatment, as well as the calculated molecular weight of the taxon in the ^12^C treatment as described in Hungate et al. (equation 10) (34). The APE of ^13^C for each taxon was then calculated by finding the difference between the molecular weights of the taxon in the ^12^C and ^13^C treatments, in reference to the background fractional abundance of ^13^C (0.01111233) (equation 12) (34). All APE estimations were then bootstrapped (1,000 draws), giving an estimate of average APE and a 95% CI for each taxon. Taxa (ASV) with a positive average APE and a lower 95% CI above zero were determined to be enriched with ^13^C and thus identified as active community members (Fig. S4).

A new data frame was created of active taxa (grouped to the genus level) and their total absolute abundance in the active community. To do so, the absolute abundance of all active (qSIP identified) ASV was summed for each individual taxa (genus level) using the normalized abundance ASV tables produced during sequencing of the heavy and medium fractions. This was done for each individual ^13^C-treated replicate core. In addition to qSIP identified ASV, these new data frame included ASV only identified in the ^13^C-acetate treatment that thus did not have an APE calculated. These ASVs were included as active if the lower 95% CI of the bootstrapped taxon (ASV)-specific WAD was greater than the upper 95% CI of the average WAD for the ^13^C treatment cores. Only heavy and medium fraction density groups were used to calculate the total absolute abundance as their measured density (temperature corrected refractive index) was above the bootstrapped WAD for the ^13^C treatments. We believe this allows us to get a more accurate representation of the abundance of the portion of the community that utilized ^13^C-acetate. To note, one core in the natural oxic–anoxic treatment is missing a medium density group due to variability in sequencing success (Table S3). We then averaged the total absolute abundance of each active taxon across the individual cores for all five wetland redox treatment groups. This gave us a final estimate of average total absolute abundance for all taxa representing the ASV identified as active.

To better assess the background abundance of active taxa in both the ^12^C- and ^13^C-treated cores, we modified and repeated the above process. Briefly, the absolute abundance of all active taxa was added across all density groups (heavy, medium, and light) in each ^12^C- and ^13^C-treated core. The average total absolute abundance across ^12^C and ^13^C replicate cores was then calculated for each wetland treatment group (Fig. S5). To note, the natural oxic–anoxic treatment also had one ^12^C labeled core missing a medium and light group, one ^12^C labeled core missing a medium and heavy group, and one ^12^C labeled core missing a light group. In addition, data are missing for one light density group in one ^13^C and one ^12^C labeled natural anoxic core and one light and one medium density group in one ^13^C labeled core in the natural oxic–anoxic treatment (Table S3).

### Statistical analysis

All statistical analysis and visualization were performed using R studio (v.4.6.1, 2022.02.1). For redox measures collected at the beginning and end of incubations, and for all edaphic measures assessed after soil core destructive sampling (GWC, pH, DOC, total Fe, and free aqueous Fe^2+^), normality was assessed using the Shapiro–Wilk normality test (alpha = 0.05, function *shapiro.test* package *stats* [v4.2.2]). All cores that received acetate (^12^C and ^13^C) were treated as replicates for assessment of redox and edaphic measures (restored anoxic: *n* = 6, restored oxic: *n* = 6, natural anoxic: *n* = 6, natural oxic: *n* = 6, natural oxic–anoxic *n* = 6). We used a two-way ANOVA (function *aov* in package *stats* [v.4.2.2]) to assess how redox (mV) and edaphic measures (GWC, pH, DOC, total Fe, and free, aqueous Fe^2+^) varied with wetland core type, redox condition, and between conditions within the wetland type cores. All interaction effects were examined. Post hoc analysis was performed using Tukey’s honestly significant difference tests with a 95% family-wise confidence level in package *stats* (v.4.2.2).

We again employed a two-way ANOVA to assess variation in measures of community diversity (number of taxa (to the individual genera level), Shannon diversity index) and size (total number of gene copies per ng DNA) for the active community (Table 2). The two-way ANOVA employed was again intended to test variation across wetland type, redox condition, and between conditions within each wetland type. Replication for the active communities corresponds to original ^13^C replicates in each acetate treatment type (restored anoxic *n* = 3, restored oxic *n* = 3, natural anoxic *n* = 2, natural oxic *n* = 3, natural oxic–anoxic *n* = 2) (Table S3). The Shannon diversity index for each replicate core’s active community was calculated using the function *diversity* in the package *vegan* (v.2.6–4) using the total absolute abundance of each taxa. Normality in data were verified (Shapiro–Wilk normality test, alpha = 0.05), and post hoc analysis was performed using Tukey’s honestly significant difference tests with a 95% family-wise confidence level (v.4.2.2).

Active community composition and differences in taxa absolute abundance between redox condition groups were also assessed. Community composition was assessed using non-metric multidimensional scaling based on Bray–Curtis dissimilarity distances (function *vegdist*, distance *bray*, package *vegan* [v.2.6–4]). Distance between communities was then assessed using a two-way PERMANOVA of wetland type and redox condition (function *adonis2* package *vegan* [v.2.6–4)]. Comparison of active abundance was performed at the phylum level (Fig. 5), between wetland redox treatments using non-parametric Kruskal–Wallis rank sum tests (function *kruskal.test* in *stats* [v.4.2.2]) and post hoc pairwise Wilcoxon rank-sum tests (function *pairwise.wilcox.test* in *stats* [v.4.2.2]).

As 161 of the 333 taxa identified (48%) were unique to just one wetland redox group, we report here the taxa found uniquely in one group or found in multiple groups (overlap) (Fig. 6; Fig. S6). This overlap was determined using function *discern_overlap* in package *ggVennDiagram* (v.1.5.2). Lists of active taxa shared between two groups or independently found in one group are listed in the supplemental material (Table S4).

## Data Availability

The sequence data generated in this work have been deposited in the National Center for Biotechnology Information Sequence Read Archive Database under BioProject number PRJNA1136864.

## SUPPLEMENTAL MATERIAL

Supplemental material aem.02161-24-s0001.docx Tables S1 to S4; Figures S1 to S7.

ASM does not own the copyrights to Supplemental Material that may be linked to, or accessed through, an article. The authors have granted ASM a non-exclusive, world-wide license to publish the Supplemental Material files. Please contact the corresponding author directly for reuse.

## References

[1] Saunois M, Stavert AR, Poulter B, Bousquet P, Canadell JG, Jackson RB, Raymond PA, Dlugokencky EJ, Houweling S, Patra PK, et al.. 2020. The global methane budget 2000–2017. Earth Syst Sci Data 12:1561–1623. doi:10.5194/essd-12-1561-2020

[2] Bridgham SD, Cadillo-Quiroz H, Keller JK, Zhuang Q. 2013. Methane emissions from wetlands: biogeochemical, microbial, and modeling perspectives from local to global scales. Glob Chang Biol 19:1325–1346. doi:10.1111/gcb.12131 23505021

[3] Mitsch WJ, Bernal B, Nahlik AM, Mander Ü, Zhang L, Anderson CJ, Jørgensen SE, Brix H. 2013. Wetlands, carbon, and climate change. Landscape Ecol 28:583–597. doi:10.1007/s10980-012-9758-8

[4] Moreno-Mateos D, Power ME, Comín FA, Yockteng R. 2012. Structural and functional loss in restored wetland ecosystems. PLoS Biol 10:e1001247. doi:10.1371/journal.pbio.1001247 22291572 PMC3265451

[5] Prasse CE, Baldwin AH, Yarwood SA. 2015. Site history and edaphic features override the influence of plant species on microbial communities in restored tidal freshwater wetlands. Appl Environ Microbiol 81:3482–3491. doi:10.1128/AEM.00038-15 25769832 PMC4407224

[6] Ballantine K, Schneider R. 2009. Fifty-five years of soil development in restored freshwater depressional wetlands. Ecol Appl 19:1467–1480. doi:10.1890/07-0588.1 19769095

[7] Craft C, Megonigal P, Broome S, Stevenson J, Freese R, Cornell J, Zheng L, Sacco J. 2003. The pace of ecosystem development of constructed Spartina alterniflora marshes freese. Ecol Appl 13:1417–1432. doi:10.1890/02-5086

[8] Brown J, Norris MD. 2018. Detecting soil and plant community changes in restored wetlands using a chronosequence approach. Wetlands Ecol Manage 26:299–314. doi:10.1007/s11273-017-9574-7

[9] Chamberlain SD, Anthony TL, Silver WL, Eichelmann E, Hemes KS, Oikawa PY, Sturtevant C, Szutu DJ, Verfaillie JG, Baldocchi DD. 2018. Soil properties and sediment accretion modulate methane fluxes from restored wetlands. Glob Chang Biol 24:4107–4121. doi:10.1111/gcb.14124 29575340

[10] Badiou P, McDougal R, Pennock D, Clark B. 2011. Greenhouse gas emissions and carbon sequestration potential in restored wetlands of the Canadian prairie pothole region. Wetlands Ecol Manage 19:237–256. doi:10.1007/s11273-011-9214-6

[11] Vanselow-Algan M, Schmidt SR, Greven M, Fiencke C, Kutzbach L, Pfeiffer E-M. 2015. High methane emissions dominated annual greenhouse gas balances 30 years after bog rewetting. Biogeosciences 12:4361–4371. doi:10.5194/bg-12-4361-2015

[12] Arias‐Ortiz A, Oikawa PY, Carlin J, Masqué P, Shahan J, Kanneg S, Paytan A, Baldocchi DD. 2021. Tidal and nontidal marsh restoration: a trade-off between carbon sequestration, methane emissions, and soil accretion. JGR Biogeosciences 126:1–22. doi:10.1029/2021JG00657337089664

[13] Malerba ME, Friess DA, Peacock M, Grinham A, Taillardat P, Rosentreter JA, Webb J, Iram N, Al-Haj AN, Macreadie PI. 2022. Methane and nitrous oxide emissions complicate the climate benefits of teal and blue carbon wetlands. One Earth 5:1336–1341. doi:10.1016/j.oneear.2022.11.003

[14] Hemes KS, Chamberlain SD, Eichelmann E, Knox SH, Baldocchi DD. 2018. Biogeochemical compromise: the high methane cost of sequestering carbon in restored wetlands. Geophys Res Lett 45:6081–6091. doi:10.1029/2018GL077747

[15] Fenstermacher DE, Rabenhorst MC, Lang MW, McCarty GW, Needelman BAD. 2014. Distribution, morphometry, and land use of delmarva bays. Wetlands (Wilmington) 34:1219–1228. doi:10.1007/s13157-014-0583-5

[16] Maietta CE, Hondula KL, Jones CN, Palmer MA. 2020. Hydrological conditions influence soil and methane-cycling microbial populations in seasonally saturated wetlands. Front Environ Sci 8:1–12. doi:10.3389/fenvs.2020.593942

[17] Lee S, McCarty GW, Lang MW, Li X. 2020. Overview of the USDA mid-Atlantic regional wetland conservation effects assessment project. J Soil Water Conserv 75:684–694. doi:10.2489/jswc.2020.00097

[18] Lang M, et al.. 2015. Effects and effectiveness of USDA wetland conservation practices in the mid-Atlantic region: a report on the conservation effects assessment project mid-Atlantic regional wetland assessment 2008-2015, p 1–169

[19] McDonough OT, Lang MW, Hosen JD, Palmer MA. 2015. Surface hydrologic connectivity between Delmarva Bay wetlands and nearby streams along a gradient of agricultural alteration. Wetlands (Wilmington) 35:41–53. doi:10.1007/s13157-014-0591-5

[20] Conrad R. 2020. Methane production in soil environments— anaerobic biogeochemistry and microbial life between flooding and desiccation. Microorganisms 8:1–12. doi:10.3390/microorganisms8060881 PMC7357154 32545191

[21] Reim A, Hernández M, Klose M, Chidthaisong A, Yuttitham M, Conrad R. 2017. Response of methanogenic microbial communities to desiccation stress in flooded and rain-fed paddy soil from Thailand. Front Microbiol 8:785. doi:10.3389/fmicb.2017.00785 28529503 PMC5418361

[22] Hernández M, Conrad R, Klose M, Ma K, Lu Y. 2017. Structure and function of methanogenic microbial communities in soils from flooded rice and upland soybean fields from Sanjiang plain, NE China. Soil Biol Biochem 105:81–91. doi:10.1016/j.soilbio.2016.11.010

[23] Hernández M, Klose M, Claus P, Bastviken D, Marotta H, Figueiredo V, Enrich-Prast A, Conrad R. 2019. Structure, function and resilience to desiccation of methanogenic microbial communities in temporarily inundated soils of the Amazon rainforest (Cunia Reserve, Rondonia). Environ Microbiol 21:1702–1717. doi:10.1111/1462-2920.14535 30680883

[24] McFarland EK, LaForgia M, Yepsen M, Whigham DF, Baldwin AH, Lang M. 2016. Plant biomass and nutrients (C, N and P) in natural, restored and prior converted depressional wetlands in the Mid-Atlantic Coastal Plain, U.S. Folia Geobot 51:267–283. doi:10.1007/s12224-016-9239-y

[25] Spadafora E, Leslie AW, Culler LE, Smith RF, Staver KW, Lamp WO. 2016. Macroinvertebrate community convergence between natural, rehabilitated, and created wetlands. Restoration Ecology 24:463–470. doi:10.1111/rec.12352

[26] Hartman WH, Richardson CJ, Vilgalys R, Bruland GL. 2008. Environmental and anthropogenic controls over bacterial communities in wetland soils. Proc Natl Acad Sci U S A 105:17842–17847. doi:10.1073/pnas.0808254105 19004771 PMC2584698

[27] Bossio DA, Fleck JA, Scow KM, Fujii R. 2006. Alteration of soil microbial communities and water quality in restored wetlands. Soil Biol Biochem 38:1223–1233. doi:10.1016/j.soilbio.2005.09.027

[28] Kluber LA, Miller JO, Ducey TF, Hunt PG, Lang M, S. Ro K. 2014. Multistate assessment of wetland restoration on CO2 and N2O emissions and soil bacterial communities. Agric, Ecosyst Environ, Appl Soil Ecol 76:87–94. doi:10.1016/j.apsoil.2013.12.014

[29] Bruland GL, Hanchey MF, Richardson CJ. 2003. Effects of agriculture and wetland restoration on hydrology, soils, and water quality of a Carolina bay complex. Wetl Ecol Manag 11:141–156. doi:10.1023/A:1024244408577

[30] Zehnder AJB, Stumn, W. 1998. Biology of anaerobic microorganisms, p 1–38. In . John Wiley & Sons Inc.

[31] Reiche M, Torburg G, Küsel K. 2008. Competition of Fe(III) reduction and methanogenesis in an acidic fen. FEMS Microbiol Ecol 65:88–101. doi:10.1111/j.1574-6941.2008.00523.x 18559015

[32] Roden EE, Wetzel RG. 2003. Competition between Fe(III)-reducing and methanogenic bacteria for acetate in iron-rich freshwater sediments. Microb Ecol 45:252–258. doi:10.1007/s00248-002-1037-9 12658519

[33] Achtnich C, Bak F, Conrad R. 1995. Competition for electron donors among nitrate reducers, ferric iron reducers, sulfate reducers, and methanogens in anoxic paddy soil. Biol Fertil Soils 19:65–72. doi:10.1007/BF00336349

[34] Hungate BA, Mau RL, Schwartz E, Caporaso JG, Dijkstra P, van Gestel N, Koch BJ, Liu CM, McHugh TA, Marks JC, Morrissey EM, Price LB. 2015. Quantitative microbial ecology through stable isotope probing. Appl Environ Microbiol 81:7570–7581. doi:10.1128/AEM.02280-15 26296731 PMC4592864

[35] Stams AJM, Teusink B, Sousa D. 2019. Edited by by A. J. Stams. Biogenesis of Hydrocarbons, p 109–123. Springer.

[36] Lyu Z, Lu Y. 2018. Metabolic shift at the class level sheds light on adaptation of methanogens to oxidative environments. ISME J 12:411–423. doi:10.1038/ismej.2017.173 29135970 PMC5776455

[37] Liu F, Conrad R. 2011. Chemolithotrophic acetogenic H2/CO2 utilization in Italian rice field soil. ISME J 5:1526–1539. doi:10.1038/ismej.2011.17 21368906 PMC3160675

[38] Mulat DG, Ward AJ, Adamsen APS, Voigt NV, Nielsen JL, Feilberg A. 2014. Quantifying contribution of synthrophic acetate oxidation to methane production in thermophilic anaerobic reactors by membrane inlet mass spectrometry. Environ Sci Technol 48:2505–2511. doi:10.1021/es403144e 24437339

[39] Lee S-H, Park J-H, Kim S-H, Yu BJ, Yoon J-J, Park H-D. 2015. Evidence of syntrophic acetate oxidation by Spirochaetes during anaerobic methane production. Bioresour Technol 190:543–549. doi:10.1016/j.biortech.2015.02.066 25739997

[40] Karakashev D, Batstone DJ, Trably E, Angelidaki I. 2006. Acetate oxidation is the dominant methanogenic pathway from acetate in the absence of Methanosaetaceae. Appl Environ Microbiol 72:5138–5141. doi:10.1128/AEM.00489-06 16820524 PMC1489330

[41] Dyksma S, Jansen L, Gallert C. 2020. Syntrophic acetate oxidation replaces acetoclastic methanogenesis during thermophilic digestion of biowaste. Microbiome 8:105. doi:10.1186/s40168-020-00862-5 32620171 PMC7334858

[42] Zheng D, Wang H-Z, Gou M, Nobu MK, Narihiro T, Hu B, Nie Y, Tang Y-Q. 2019. Identification of novel potential acetate-oxidizing bacteria in thermophilic methanogenic chemostats by DNA stable isotope probing. Appl Microbiol Biotechnol 103:8631–8645. doi:10.1007/s00253-019-10078-9 31418053

[43] Yi Y, Wang H, Chen Y, Gou M, Xia Z, Hu B, Nie Y, Tang Y. 2020. Identification of novel butyrate- and acetate-oxidizing bacteria in butyrate-fed mesophilic anaerobic chemostats by DNA-based stable isotope probing. Microb Ecol 79:285–298. doi:10.1007/s00248-019-01400-z 31263981

[44] Westerholm M, Müller B, Singh A, Karlsson Lindsjö O, Schnürer A. 2018. Detection of novel syntrophic acetate-oxidizing bacteria from biogas processes by continuous acetate enrichment approaches. Microb Biotechnol 11:680–693. doi:10.1111/1751-7915.13035 29239113 PMC6011928

[45] Song C, Li W, Cai F, Liu G, Chen C. 2021. Anaerobic and microaerobic pretreatment for improving methane production from paper waste in anaerobic digestion. Front Microbiol 12:688290. doi:10.3389/fmicb.2021.688290 34295321 PMC8290346

[46] Ho A, Kerckhof F-M, Luke C, Reim A, Krause S, Boon N, Bodelier PLE. 2013. Conceptualizing functional traits and ecological characteristics of methane-oxidizing bacteria as life strategies. Environ Microbiol Rep 5:335–345. doi:10.1111/j.1758-2229.2012.00370.x 23754714

[47] Ho A, van den Brink E, Reim A, Krause SMB, Bodelier PLE. 2015. Recurrence and frequency of disturbance have cumulative effect on methanotrophic activity, abundance, and community structure. Front Microbiol 6:1493. doi:10.3389/fmicb.2015.01493 26779148 PMC4700171

[48] Zhang L, Adams JM, Dumont MG, Li Y, Shi Y, He D, He J-S, Chu H. 2019. Distinct methanotrophic communities exist in habitats with different soil water contents. Soil Biol Biochem 132:143–152. doi:10.1016/j.soilbio.2019.02.007

[49] Guerrero-Cruz S, Vaksmaa A, Horn MA, Niemann H, Pijuan M, Ho A. 2021. Methanotrophs: discoveries, environmental relevance, and a perspective on current and future applications. Front Microbiol 12:678057. doi:10.3389/fmicb.2021.678057 34054786 PMC8163242

[50] Epron D, Plain C, Ndiaye F-K, Bonnaud P, Pasquier C, Ranger J. 2016. Effects of compaction by heavy machine traffic on soil fluxes of methane and carbon dioxide in a temperate broadleaved forest. For Ecol Manage 382:1–9. doi:10.1016/j.foreco.2016.09.037

[51] Frey B, Niklaus PA, Kremer J, Lüscher P, Zimmermann S. 2011. Heavy-machinery traffic impacts methane emissions as well as methanogen abundance and community structure in oxic forest soils. Appl Environ Microbiol 77:6060–6068. doi:10.1128/AEM.05206-11 21742929 PMC3165404

[52] Zhu R, Wang DH, Zheng Y, Zou H, Fu SF. 2022. Understanding the mechanisms behind micro-aeration to enhance anaerobic digestion of corn straw. Fuel (Lond) 318:123604. doi:10.1016/j.fuel.2022.123604

[53] Fu S, Lian S, Angelidaki I, Guo R. 2023. Micro-aeration: an attractive strategy to facilitate anaerobic digestion. Trends Biotechnol 41:714–726. doi:10.1016/j.tibtech.2022.09.008 36216713

[54] Komagata K, Takao I, Yamada Y. 2014. The Prokaryotes: Alphaproteobacteria and Betaproteobacteria. Springer.

[55] Kotsyurbenko, OR, Glagolev, MV, Merkel, AY, Sabrekov, AF, Terentieva, IE. 2019. Methanogenesis in soils, wetlands, and peat, p 211–228. In Stams, A, Sousa D (ed), Biogenesis of hydrocarbons. Handbook of hydrocarbon and lipid microbiology. Springer Cham.

[56] Brinson MM, Eckles SD. 2011. U.S. Department of Agriculture conservation program and practice effects on wetland ecosystem services: a synthesis. Ecol Appl 21. doi:10.1890/09-0627.1

[57] Mueller SC, Stolzy LH, Fick GW. 1985. Constructing and screening platinum microelectrodes for measuring soil redox potential. Soil Sci 139:558–560. doi:10.1097/00010694-198506000-00013

[58] Holmgren GGS. 1967. A rapid citrate‐dithionite extractable iron procedure. Soil Science Soc of Amer J 31:210–211. doi:10.2136/sssaj1967.03615995003100020020x

[59] Roy Chowdhury T, Mitsch WJ, Dick RP. 2014. Seasonal methanotrophy across a hydrological gradient in a freshwater wetland. Ecol Eng 72:116–124. doi:10.1016/j.ecoleng.2014.08.015

[60] Alessi DS, Walsh DM, Fein JB. 2011. Uncertainties in determining microbial biomass C using the chloroform fumigation–extraction method. Chem Geol 280:58–64. doi:10.1016/j.chemgeo.2010.10.014

[61] Horwath WR, Paul EA. 1994. Microbial biomass, p 753. In Methods of soil analysis, Part 2. Microbiological and biochemical properties. Soil Science Society of America.

[62] Bowen H, Maul JE, Cavigelli MA, Yarwood S. 2020. Denitrifier abundance and community composition linked to denitrification activity in an agricultural and wetland soil. Agric, Ecosyst Environ, Appl Soil Ecol 151:103521. doi:10.1016/j.apsoil.2020.103521

[63] Neufeld JD, Vohra J, Dumont MG, Lueders T, Manefield M, Friedrich MW, Murrell JC. 2007. DNA stable-isotope probing. Nat Protoc 2:860–866. doi:10.1038/nprot.2007.109 17446886

[64] Buckley DH, Huangyutitham V, Hsu SF, Nelson TA. 2007. Stable isotope probing with 15N achieved by disentangling the effects of genome G+C content and isotope enrichment on DNA density. Appl Environ Microbiol 73:3189–3195. doi:10.1128/AEM.02609-06 17369331 PMC1907112

[65] Beulig F, Heuer VB, Akob DM, Viehweger B, Elvert M, Herrmann M, Hinrichs K-U, Küsel K. 2015. Carbon flow from volcanic CO2 into soil microbial communities of a wetland mofette. ISME J 9:746–759. doi:10.1038/ismej.2014.148 25216086 PMC4331572

[66] Caporaso JG, Lauber CL, Walters WA, Berg-Lyons D, Lozupone CA, Turnbaugh PJ, Fierer N, Knight R. 2011. Global patterns of 16S rRNA diversity at a depth of millions of sequences per sample. Proc Natl Acad Sci U S A 108 Suppl 1:4516–4522. doi:10.1073/pnas.1000080107 20534432 PMC3063599

[67] Hargreaves SK, Roberto AA, Hofmockel KS. 2013. Reaction- and sample-specific inhibition affect standardization of qPCR assays of soil bacterial communities. Soil Biol Biochem 59:89–97. doi:10.1016/j.soilbio.2013.01.007

[68] Callahan BJ, McMurdie PJ, Rosen MJ, Han AW, Johnson AJA, Holmes SP. 2016. DADA2: high-resolution sample inference from Illumina amplicon data. Nat Methods 13:581–583. doi:10.1038/nmeth.3869 27214047 PMC4927377

[69] McMurdie PJ, Holmes SP. 2013. Phyloseq: an R package for reproducible interactive analysis and graphics of microbiome census data. PLoS ONE 8:e61217. doi:10.1371/journal.pone.0061217 23630581 PMC3632530

[70] Oksanen J, et al.. 2022. Vegan: community ecology package. R package version 2.6-2

[71] Epp Schmidt D, Dlott G, Cavigelli M, Yarwood S, Maul JE. 2022. Soil microbiomes in three farming systems more affected by depth than farming system. Agric, Ecosyst Environ, Appl Soil Ecol 173:104396. doi:10.1016/j.apsoil.2022.104396

[72] Jian C, Luukkonen P, Yki-Järvinen H, Salonen A, Korpela K. 2020. Quantitative PCR provides a simple and accessible method for quantitative microbiota profiling. PLoS One 15:e0227285. doi:10.1371/journal.pone.0227285 31940382 PMC6961887

