# Supplementary material for: Comparative assessment of a restored and natural wetland using 13C-DNA SIP reveals a higher potential for methane production in the restored wetland
Source: Appl Environ Microbiol. 2025 Feb 6;91(3):e02161-24. doi: 10.1128/aem.02161-24 (PMC11921397; doi:10.1128/aem.02161-24)
Supplement: Supplemental material — Tables S1 to S4; Figures S1 to S7. [file aem.02161-24-s0001.docx]

**Supplementary Material**

**Comparative assessment of a restored and natural wetland using ^13^C-DNA SIP reveals a higher potential for methane production in the restored wetland**

Nora Hamovit^1*^, Taniya RoyChowdhury^2↟^, Denise M. Akob^3^, Xuesong Zhang^4^, Gregory McCarty^4^, Stephanie Yarwood^2^

^1^Department of Biological Sciences, University of Maryland, College Park, MD

^2^Department of Environmental Science and Technology, University of Maryland, College Park, MD

**^↟^**Current affiliation: Woodwell Climate Research Center, Falmouth, MA

^3^Geology, Energy & Minerals Science Center, U.S. Geological Survey, Reston, VA

^4^Agricultural Research Center, United States Department of Agriculture, Beltsville, MD

*corresponding author email: [nhamovit@umd.edu](mailto:nhamovit@umd.edu)

**Table of Contents**

[Supplementary Tables 3](#_Toc186202071)

[Table S1. CH_4_ production rates from the last 14 days of the pre-incubations. All data from natural cores was below the gas chromatograph detection limit (0.0001% CH_4_). Data was collected from the headspace of cores 14 days before incubations began (day -14), 8 days before incubations began (-8), and the day incubations began (day 0). Rates are reported as µmoles CH_4_ g^-1^ oxic weight soil day^-1^. Statistics described the fit of the linear regression. na=not available. 3](#_Toc186202072)

[Table S2. The total number of ASV and total absolute abundance (qPCR corrected relative abundance) calculated for the whole community in each sample sequenced. Wetland and redox treatment are indicated as well as density group and amendment type.. 4](#_Toc186202073)

[Table S3. Number of replicates of each density fraction used for qSIP analysis from both the ^12^C-and ^13^C-acetate amended cores. 6](#_Toc186202074)

[Table S4. List of taxa unique to each active community, or uniquely found in two active communities. 7](#_Toc186202075)

[Supplemental Figures 13](#_Toc186202076)

[Figure S1. Diagram of incubation design. a.) Soil cores were collected from wetlands in intact glass sleeves using custom designed soil corer. Soil cores were 15 cm deep and 2.54 cm in diameter. b.) Glass sleeves measured 20 cm, leaving 5 cm of headspace above the 15 cm soil core. Cores were sealed with air-tight caps. Redox probes were installed at 5 cm, 7.5 cm, and 15 cm depth in the restored cores and 5 cm and 10 cm depth in the natural cores. A septum at the bottom of the core stand allowed saturation to be maintained. Soil drainage occurred out of the bottom of the core. 13](#_Toc186202077)

[Figure S2. Schematic represents anticipated change in redox (mV) at varying depths for each of the redox condition groups (y-axis facet) and the acetate additions (x-axis facet). Within each group and acetate addition type there are three replicate cores. Depth of redox reading within the core is indicated by color. The anticipated first redox measurements are from the pre-incubation (day -14 and -7), followed by a measurement made at the initiation (day 0) of the incubations. Vertical line at day 0 indicates beginning of incubation. 14](#_Toc186202078)

[Figure S3. Average percent (%) of total recovered DNA in each of the 12 fractions plotted by the average density (g/µL) of corresponding fraction. Fractions to the left of the vertical black line at 1.69 g/µL indicates light density fractions. Fractions to the left of vertical black line at 1.75 g/µL indicate medium density fractions. Fractions to the right of vertical black line at 1.75 g/µL indicate the heavy density fraction. Vertical error bars indicate the standard error of mean for % DNA in fractions. Horizontal error bars indicate the standard error of mean for density of each fraction. 15](#_Toc186202079)

[Figure S4. Average atom percent excess (APE) of ^13^C in DNA in each class, grouped by phyla and characterized by wetland type and redox condition. Positive APE represents ^13^C incorporation into the DNA. Each dot represents the average APE of a class within the phylum. Horizontal bars show average bootstrapped 95% confidence intervals around each class’s mean APE. Phyla are color coded. Note independent x and y axis scale 16](#_Toc186202080)

[Figure S5. Average absolute abundance (relative abundance normalized by gene copies/ng of DNA) of all active taxa in both the 12C and 13C labeled cores (x-axis). Taxa are grouped by phylum and by genera for methanogens in the phylum *Euryarchaeota*. Taxa are listed alphabetically by phylum and facetted by kingdom (Archaea and Bacteria). Size of the circle corresponds to average absolute abundance in the wetland’s different redox treatment communities. Color indicates wetland type and redox condition. Conditions are listed left to right: natural oxic, natural oxic-anoxic, natural anoxic, restored oxic, and restored anoxic. 17](#_Toc186202081)

[Figure S6. Average concentration of Fe forms in each core plotted according to corresponding final redox (mV) measurement in the core. The left panel plots average Fe^2+^ concentration (blue) in each core and right panel plots average total Fe concentration (black) in each core. Horizontal bars correspond to standard deviation. Total Fe and Fe^2+^ concentrations positively corelate (linear regression, p = 0.002, R^2^ = 0.29). Average Fe^2+^ concentrations were higher in the restored wetland (ANOVA, p = 0.004), as were average concentrations of total Fe (ANOVA, p = 0.007). Note independent y axis scale. NA indicates no measurement taken. 18](#_Toc186202082)

[Figure S7. Venn Diagram showing the number of taxa that co-occur, or are independent, in the wetland redox conditions. Outline color represents the redox condition. The background color represents the number of taxa in that section. A higher count of taxa is indicated by a more yellow background, while a lower count is indicated by a greener background. The number in each section is the number of taxa, percent total taxa is shown below (x%). 19](#_Toc186202083)

# Supplementary Tables

## Table S1. CH_4_ production rates from the last 14 days of the pre-incubations. All data from natural cores was below the gas chromatograph detection limit (0.0001% CH_4_). Data was collected from the headspace of cores 14 days before incubations began (day -14), 8 days before incubations began (-8), and the day incubations began (day 0). Rates are reported as µmoles CH_4_ g^-1^ oxic weight soil day^-1^. Statistics described the fit of the linear regression. na=not available.

| **Redox status** | **Replicate** | **Rate** | **p-value** | **R^2^** |
| --- | --- | --- | --- | --- |
| **Restored oxic** | 1 | 0.037 ± 0.006 | 0.109 | 0.94 |
|  | 2 | 0.000 ± 0.000 | 0.628 | -0.39 |
|  | 3 | 0.003 ± 0.005 | 0.660 | -0.48 |
|  | 4 | -0.001 ± 0.019 | 0.959 | -0.99 |
|  | 5 | 0.001 ± na | na | na |
|  | 6 | 0.000 ± 0.000 | 0.090 | 0.96 |
|  | 7 | 0.001 ± 0.001 | 0.359 | 0.43 |
|  | 8 | 0.003 ± 0.004 | 0.634 | -0.41 |
|  | 9 | 0.000 ± 0.000 | 0.433 | 0.21 |
| **Restored anoxic** | 1 | 0.002 ± 0.001 | 0.338 | 0.49 |
|  | 2 | 0.017 ± 0.001 | 0.025 | 1.00 |
|  | 3 | -0.022 ± 0.012 | 0.322 | 0.53 |
|  | 4 | 0.028 ± 0.014 | 0.297 | 0.60 |
|  | 5 | 0.001 ± 0.002 | 0.791 | -0.79 |
|  | 6 | 0.028 ± 0.010 | 0.207 | 0.80 |
|  | 7 | 0.000 ± 0.000 | 0.184 | 0.84 |
|  | 8 | 0.000 ± 0.001 | 0.687 | -0.55 |
|  | 9 | -0.023 ± 0.018 | 0.412 | 0.27 |

## Table S2. The total number of ASV and total absolute abundance (qPCR corrected relative abundance) calculated for the whole community in each sample sequenced. Wetland and redox treatment are indicated as well as density group and amendment type.

| **Wetland and Redox Status** | **Amendment** | **Density**  **Group** | **No. ASV** | **Total Absolute Abundance**  **(gene copies ng^-1^ DNA)** |
| --- | --- | --- | --- | --- |
| Natural oxic | ^12^C-acetate | heavy | 1508 | 121993.5825 |
|  |  | heavy | 1275 | 10972.43664 |
|  |  | heavy | 1371 | 1261.54264 |
|  |  | medium | 1019 | 11249.80072 |
|  |  | medium | 797 | 12548.22597 |
|  |  | medium | 304 | 2003.67239 |
|  |  | light | 1124 | 15606.3428 |
|  |  | light | 809 | 30132.76715 |
|  |  | light | 1300 | 14619.05825 |
|  | ^13^C-acetate | heavy | 1010 | 1003.74122 |
|  |  | heavy | 1371 | 134.33 |
|  |  | heavy | 839 | 41444.08114 |
|  |  | medium | 895 | 165835.9163 |
|  |  | medium | 1126 | 6667.67428 |
|  |  | light | 1650 | 36826.60509 |
|  |  | light | 628 | 118794.141 |
|  |  | light | 409 | 59778.60268 |
| Natural oxic-anoxic | ^12^C-acetate | heavy | 640 | 1596.24043 |
|  |  | heavy | 1277 | 9329.30199 |
|  |  | medium | 527 | 1050.64712 |
|  |  | light | 819 | 22132.35432 |
|  | ^13^C-acetate | heavy | 550 | 351.07919 |
|  |  | heavy | 193 | 81.71502 |
|  |  | medium | 633 | 9901.14583 |
|  |  | light | 883 | 2523.16423 |
| Natural anoxic | ^12^C-acetate | heavy | 249 | 10.6541 |
|  |  | heavy | 1109 | 1789.53123 |
|  |  | heavy | 615 | 1312.29002 |
|  |  | medium | 326 | 4747.95704 |
|  |  | medium | 1013 | 4625.17907 |
|  |  | medium | 975 | 17800.48717 |
|  |  | light | 67 | 162.97156 |
|  |  | light | 1328 | 42633.90137 |
|  | ^13^C-acetate | heavy | 1443 | 4781.86853 |
|  |  | heavy | 1680 | 8310.85668 |
|  |  | medium | 1715 | 822.09291 |
|  |  | medium | 1840 | 2332.04641 |
|  |  | light | 740 | 89732.54784 |
|  |  | light | 1183 | 23624.56776 |

**Table S3*, continued***

| **Wetland and Redox Status** | **Amendment** | **Density**  **Group** | **No. ASV** | **Total Absolute Abundance**  **(gene copies ng^-1^ DNA)** |
| --- | --- | --- | --- | --- |
| Restored oxic | ^12^C-acetate | heavy | 433 | 7244.73351 |
|  |  | heavy | 1027 | 470.49105 |
|  |  | heavy | 214 | 311.84097 |
|  |  | medium | 1830 | 22871.46747 |
|  |  | medium | 318 | 9248.51484 |
|  |  | medium | 215 | 17.51088 |
|  |  | light | 2389 | 699010.8592 |
|  |  | light | 1372 | 25689.85266 |
|  |  | light | 1173 | 89326.02353 |
|  | ^13^C-acetate | heavy | 426 | 20283.76689 |
|  |  | heavy | 107 | 196.75574 |
|  |  | heavy | 925 | 7173.62554 |
|  |  | medium | 1352 | 217.09739 |
|  |  | medium | 529 | 2854.89552 |
|  |  | medium | 1032 | 29694.3945 |
|  |  | light | 2092 | 415590.3801 |
|  |  | light | 685 | 42629.90167 |
|  |  | light | 1305 | 307325.8437 |
| Restored anoxic | ^12^C-acetate | heavy | 1268 | 34084.60707 |
|  |  | heavy | 1378 | 54812.45683 |
|  |  | heavy | 1519 | 5946.22391 |
|  |  | medium | 756 | 8761.57416 |
|  |  | medium | 1964 | 580550.4757 |
|  |  | medium | 1723 | 14059.46668 |
|  |  | light | 1124 | 220678.5312 |
|  |  | light | 1889 | 301803.921 |
|  |  | light | 2159 | 14630.91822 |
|  | ^13^C-acetate | heavy | 1249 | 35273.69504 |
|  |  | heavy | 1037 | 77605.55628 |
|  |  | heavy | 1766 | 5329.40492 |
|  |  | medium | 580 | 2394.76111 |
|  |  | medium | 1287 | 8988.22276 |
|  |  | medium | 1278 | 4736.24658 |
|  |  | light | 1049 | 518400.4883 |
|  |  | light | 1106 | 301803.921 |
|  |  | light | 1362 | 233553.8337 |

## Table S3. Number of replicates of each density fraction used for qSIP analysis from both the ^12^C-and ^13^C-acetate amended cores.

|  |  | **^12^C-Acetate** | | | **^13^C-Acetate** | | |
| --- | --- | --- | --- | --- | --- | --- | --- |
| Type | Redox status | Heavy | Medium | Light | Heavy | Medium | Light |
|  |  | # | # | # | # | # | # |
| **Natural** | **anoxic** | 3 | 3 | 2 | 2 | 2 | 2 |
|  | **oxic** | 3 | 3 | 3 | 3 | 2 | 3 |
|  | **oxic-anoxic** | 2 | 1 | 1 | 2 | 1 | 1 |
| **Restored** | **anoxic** | 3 | 3 | 3 | 3 | 3 | 3 |
|  | **oxic** | 3 | 3 | 3 | 3 | 3 | 3 |

## Table S4. List of taxa unique to each active community, or uniquely found in two active communities.

| **Taxa unique to restored oxic** **active community** |
| --- |
| Actinobacteria Actinobacteria Catenulisporales Catenulisporaceae Catenulispora |
| Actinobacteria Actinobacteria Corynebacteriales Corynebacteriaceae Corynebacterium_1 |
| Actinobacteria Thermoleophilia Gaiellales Gaiellaceae Gaiella |
| Armatimonadetes Fimbriimonadia Fimbriimonadales Fimbriimonadaceae NA |
| Bacteroidetes Bacteroidia Sphingobacteriales Lentimicrobiaceae NA |
| Chloroflexi Anaerolineae Anaerolineales Anaerolineaceae NA |
| Chloroflexi Ktedonobacteria Ktedonobacterales Ktedonobacteraceae G12-WMSP1 |
| Chloroflexi Ktedonobacteria Ktedonobacterales Ktedonobacteraceae Ktedonobacter |
| Dependentiae Babeliae Babeliales UBA12411 NA |
| Fibrobacteres Fibrobacteria Fibrobacterales TG3 NA |
| Firmicutes Bacilli Bacillales Bacillaceae Bacillus |
| Firmicutes Bacilli Bacillales Bacillaceae NA |
| Firmicutes Bacilli Bacillales Thermoactinomycetaceae Shimazuella |
| Firmicutes Clostridia Clostridiales Family_XI Finegoldia |
| Firmicutes Clostridia Clostridiales Clostridiaceae_1 Clostridium_sensu_stricto_10 |
| Firmicutes Clostridia Clostridiales Ruminococcaceae Ruminiclostridium_1 |
| Gemmatimonadetes Gemmatimonadetes Gemmatimonadales Gemmatimonadaceae Gemmatirosa |
| Lentisphaerae Lentisphaeria Victivallales PRD18C08 NA |
| Patescibacteria ABY1 Candidatus_Falkowbacteria NA NA |
| Patescibacteria CPR2 NA NA NA |
| Patescibacteria Gracilibacteria Candidatus_Peribacteria NA NA |
| Patescibacteria Parcubacteria Candidatus_Zambryskibacteria NA NA |
| Planctomycetes Phycisphaerae Pla1_lineage NA NA |
| Planctomycetes Planctomycetacia Pirellulales Pirellulaceae Pirellula |
| Proteobacteria Alphaproteobacteria Rhizobiales Beijerinckiaceae Rhodoblastus |
| Proteobacteria Alphaproteobacteria Rhizobiales Hyphomicrobiaceae NA |
| Proteobacteria Alphaproteobacteria Rhizobiales Xanthobacteraceae Bradyrhizobium |
| Proteobacteria Alphaproteobacteria Rhizobiales Xanthobacteraceae GAS113 |
| Proteobacteria Alphaproteobacteria Elsterales Elsteraceae NA |
| Proteobacteria Alphaproteobacteria Reyranellales Reyranellaceae NA |
| Proteobacteria Deltaproteobacteria Bdellovibrionales Bdellovibrionaceae Bdellovibrio |
| Proteobacteria Gammaproteobacteria Betaproteobacteriales Burkholderiaceae Cupriavidus |
| Proteobacteria Gammaproteobacteria Betaproteobacteriales Methylophilaceae MM1 |
| Proteobacteria Gammaproteobacteria Betaproteobacteriales Nitrosomonadaceae Ellin6067 |
| Proteobacteria Gammaproteobacteria Betaproteobacteriales Nitrosomonadaceae mle1-7 |
| Proteobacteria Gammaproteobacteria Betaproteobacteriales A21b NA |
| Proteobacteria Gammaproteobacteria Betaproteobacteriales NA NA |
| Proteobacteria Gammaproteobacteria Diplorickettsiales Diplorickettsiaceae Diplorickettsia |
| Proteobacteria Gammaproteobacteria Pseudomonadales Moraxellaceae Enhydrobacter |
| Proteobacteria Gammaproteobacteria Pseudomonadales Pseudomonadaceae Pseudomonas |
| Rokubacteria NC10 Rokubacteriales NA NA |
| Verrucomicrobia Verrucomicrobiae Chthoniobacterales Chthoniobacteraceae Candidatus_Udaeobacter |
| Verrucomicrobia Verrucomicrobiae Pedosphaerales Pedosphaeraceae Pedosphaera |
| Verrucomicrobia Verrucomicrobiae Verrucomicrobiales Rubritaleaceae Luteolibacter |

| **Taxa unique to the restored anoxic active community** |
| --- |
| Thaumarchaeota Nitrososphaeria Nitrosotaleales Nitrosotaleaceae Candidatus_Nitrosotalea |
| Actinobacteria Acidimicrobiia Microtrichales Ilumatobacteraceae NA |
| Actinobacteria Actinobacteria Frankiales Geodermatophilaceae Blastococcus |
| Actinobacteria Actinobacteria Frankiales Geodermatophilaceae Geodermatophilus |
| Actinobacteria Actinobacteria Frankiales NA NA |
| Actinobacteria Actinobacteria Catenulisporales Actinospicaceae Actinospica |
| Actinobacteria Actinobacteria Micrococcales Intrasporangiaceae Oryzihumus |
| Actinobacteria Actinobacteria Micrococcales Micrococcaceae Micrococcus |
| Actinobacteria Actinobacteria Streptomycetales Streptomycetaceae Streptomyces |
| Actinobacteria Actinobacteria Streptosporangiales Streptosporangiaceae Microbispora |
| Actinobacteria Actinobacteria Streptosporangiales Streptosporangiaceae Streptosporangium |
| Actinobacteria MB-A2-108 NA NA NA |
| Bacteroidetes Bacteroidia Chitinophagales Chitinophagaceae Ferruginibacter |
| Bacteroidetes Bacteroidia Chitinophagales Chitinophagaceae Sediminibacterium |
| Bacteroidetes Ignavibacteria Ignavibacteriales Ignavibacteriaceae Ignavibacterium |
| Chlamydiae Chlamydiae Chlamydiales Parachlamydiaceae NA |
| Chloroflexi Anaerolineae RBG-13-54-9 NA NA |
| Chloroflexi Chloroflexia Thermomicrobiales Thermomicrobiaceae Sphaerobacter |
| Chloroflexi Chloroflexia NA NA NA |
| Chloroflexi Ktedonobacteria Ktedonobacterales Ktedonobacteraceae 1959-1 |
| Cyanobacteria Sericytochromatia NA NA NA |
| Firmicutes Bacilli Bacillales Pasteuriaceae Pasteuria |
| Firmicutes Clostridia Clostridiales Peptostreptococcaceae Clostridioides |
| Firmicutes Clostridia NA NA NA |
| Firmicutes Negativicutes Selenomonadales Veillonellaceae Anaerosinus |
| Firmicutes Negativicutes Selenomonadales Veillonellaceae Pelosinus |
| Firmicutes Clostridia Clostridiales Ruminococcaceae Anaerobacterium |
| Nitrospirae Nitrospira Nitrospirales Nitrospiraceae Nitrospira |
| Patescibacteria Berkelbacteria NA NA NA |
| Patescibacteria Parcubacteria Candidatus_Azambacteria NA NA |
| Planctomycetes Phycisphaerae MSBL9 NA NA |
| Planctomycetes Planctomycetacia Planctomycetales NA NA |
| Proteobacteria Alphaproteobacteria Rhizobiales Beijerinckiaceae Microvirga |
| Proteobacteria Alphaproteobacteria Rhizobiales Hyphomicrobiaceae Pedomicrobium |
| Proteobacteria Alphaproteobacteria Rhizobiales Amb-16S-1323 NA |
| Proteobacteria Deltaproteobacteria Desulfovibrionales Desulfovibrionaceae Desulfovibrio |
| Proteobacteria Deltaproteobacteria Deltaproteobacteria_Incertae_Sedis Syntrophorhabdaceae Syntrophorhabdus Syntrophorhabdus |
| Proteobacteria Gammaproteobacteria Gammaproteobacteria_Incertae_Sedis Unknown_Family Candidatus_Berkiella |
| Proteobacteria Gammaproteobacteria Xanthomonadales Rhodanobacteraceae Dokdonella |
| Verrucomicrobia Verrucomicrobiae Chthoniobacterales Chthoniobacteraceae NA |
| Verrucomicrobia Verrucomicrobiae NA NA NA |
| Verrucomicrobia Verrucomicrobiae Opitutales Puniceicoccaceae Verruc-01 |

| **Taxa unique to the natural oxic** **active community** |
| --- |
| Actinobacteria Actinobacteria Corynebacteriales Nocardiaceae Skermania |
| Bacteroidetes Bacteroidia NA NA NA |
| Chloroflexi Anaerolineae SJA-15 NA NA |
| Elusimicrobia Lineage_IIb NA NA NA |
| Fibrobacteres Fibrobacteria Fibrobacterales possible_family_01 NA |
| Firmicutes Bacilli Bacillales Family_XI Gemella |
| Firmicutes Bacilli Lactobacillales Streptococcaceae Streptococcus |
| Firmicutes Clostridia Thermoanaerobacterales Thermoanaerobacteraceae NA |
| Patescibacteria ABY1 Candidatus_Kerfeldbacteria NA NA |
| Planctomycetes Phycisphaerae MSBL9 GWC2-45-44 NA |
| Proteobacteria Alphaproteobacteria Rhizobiales Xanthobacteraceae Rhodoplanes |
| Proteobacteria Alphaproteobacteria Rickettsiales SM2D12 NA |
| Proteobacteria Deltaproteobacteria Desulfobacterales Desulfobulbaceae Desulforhopalus |
| Proteobacteria Deltaproteobacteria Myxococcales MSB-4B10 NA |
| Proteobacteria Deltaproteobacteria Oligoflexales Oligoflexaceae Silvanigrella |
| Proteobacteria Gammaproteobacteria Betaproteobacteriales Neisseriaceae Neisseria |
| Proteobacteria Gammaproteobacteria Pseudomonadales Moraxellaceae Acinetobacter |
| Proteobacteria Gammaproteobacteria Salinisphaerales Solimonadaceae Nevskia |
| Proteobacteria Gammaproteobacteria Xanthomonadales Rhodanobacteraceae Frateuria |
| Spirochaetes Leptospirae Leptospirales Leptospiraceae RBG-16-49-21 |
| Spirochaetes Spirochaetia Spirochaetales Spirochaetaceae NA |
| Verrucomicrobia Verrucomicrobiae Methylacidiphilales Methylacidiphilaceae NA |
|  |
| **Taxa unique to the natural anoxic active community** |
| Euryarchaeota Methanomicrobia Methanocellales Methanocellaceae Methanocella |
| Thaumarchaeota Nitrososphaeria Nitrososphaerales Nitrososphaeraceae NA |
| Acidobacteria Acidobacteriia Acidobacteriales Acidobacteriaceae_(Subgroup_1) Acidipila |
| Acidobacteria Acidobacteriia Acidobacteriales Acidobacteriaceae_(Subgroup_1) Edaphobacter |
| Actinobacteria Actinobacteria Micrococcales Microbacteriaceae Galbitalea |
| Actinobacteria Actinobacteria Streptomycetales Streptomycetaceae Kitasatospora |
| Actinobacteria Actinobacteria Streptosporangiales Streptosporangiaceae Herbidospora |
| Bacteroidetes Bacteroidia Chitinophagales Chitinophagaceae Chitinophaga |
| Bacteroidetes Bacteroidia Sphingobacteriales S15A-MN91 NA |
| Bacteroidetes Bacteroidia Sphingobacteriales Sphingobacteriaceae Pedobacter |
| Bacteroidetes Bacteroidia Sphingobacteriales NA NA |
| Bacteroidetes Ignavibacteria SJA-28 NA NA |
| Chlamydiae Chlamydiae Chlamydiales Parachlamydiaceae Candidatus_Protochlamydia |
| Chlamydiae Chlamydiae Chlamydiales Simkaniaceae Candidatus_Rhabdochlamydia |
| Chloroflexi Anaerolineae SBR1031 NA NA |
| Chloroflexi Anaerolineae NA NA NA |
| Chloroflexi Chloroflexia Thermomicrobiales JG30-KF-CM45 NA |
| Chloroflexi Dehalococcoidia RBG-13-46-9 NA NA |
| Chloroflexi Ktedonobacteria Ktedonobacterales Ktedonobacteraceae JG30a-KF-32 |
| Dependentiae Babeliae Babeliales UBA12409 NA |
| Firmicutes Bacilli Bacillales Planococcaceae Chungangia |
| Firmicutes Bacilli Bacillales Thermoactinomycetaceae NA |
| Firmicutes Clostridia Clostridiales Clostridiaceae_1 Clostridium_sensu_stricto_8 |
| Patescibacteria ABY1 Candidatus_Magasanikbacteria NA NA |
| Proteobacteria Alphaproteobacteria Acetobacterales Acetobacteraceae Acidisoma |
| Proteobacteria Alphaproteobacteria Acetobacterales Acetobacteraceae Acidisphaera |
| Proteobacteria Alphaproteobacteria Rhizobiales Beijerinckiaceae Methylobacterium |
| Proteobacteria Alphaproteobacteria Rhizobiales Beijerinckiaceae NA |
| Proteobacteria Alphaproteobacteria Rhizobiales Devosiaceae Devosia |
| Proteobacteria Alphaproteobacteria Rhizobiales Rhizobiaceae Allorhizobium-Neorhizobium-Pararhizobium-Rhizobium |
| Proteobacteria Alphaproteobacteria Caulobacterales Caulobacteraceae Phenylobacterium |
| Proteobacteria Alphaproteobacteria Rickettsiales Mitochondria NA |
| Proteobacteria Deltaproteobacteria Myxococcales Blfdi19 NA |
| Proteobacteria Deltaproteobacteria Myxococcales Polyangiaceae Aetherobacter |
| Proteobacteria Deltaproteobacteria Myxococcales mle1-27 NA |
| Proteobacteria Deltaproteobacteria SAR324_clade(Marine_group_B) NA NA |
| Proteobacteria Gammaproteobacteria Betaproteobacteriales Burkholderiaceae Massilia |
| Proteobacteria Gammaproteobacteria Betaproteobacteriales Burkholderiaceae Polaromonas |
| Proteobacteria Gammaproteobacteria Betaproteobacteriales Burkholderiaceae Variovorax |
| Proteobacteria Gammaproteobacteria Betaproteobacteriales Gallionellaceae Candidatus_Nitrotoga |
| Proteobacteria Gammaproteobacteria Betaproteobacteriales Gallionellaceae NA |
| Proteobacteria Gammaproteobacteria Coxiellales Coxiellaceae Coxiella |
| Proteobacteria Gammaproteobacteria Diplorickettsiales Diplorickettsiaceae Aquicella |
| Proteobacteria Gammaproteobacteria Enterobacteriales Enterobacteriaceae Enterobacter |
| Proteobacteria Gammaproteobacteria Gammaproteobacteria_Incertae_Sedis Unknown_Family Candidatus_Ovatusbacter |
| Proteobacteria Gammaproteobacteria Xanthomonadales Xanthomonadaceae Stenotrophomonas |
| Spirochaetes Spirochaetia Brevinematales Brevinemataceae Brevinema |
| Verrucomicrobia Verrucomicrobiae Chthoniobacterales Terrimicrobiaceae Terrimicrobium |

| **Taxa unique to the natural oxic-anoxic** **active community** |
| --- |
| Acidobacteria Acidobacteriia Solibacterales Solibacteraceae_(Subgroup_3) AKIW659 |
| Proteobacteria Deltaproteobacteria Myxococcales P3OB-42 NA |

| **Taxa unique to restored oxic** **and anoxic active communities** |
| --- |
| Euryarchaeota Thermoplasmata Methanomassiliicoccales Methanomassiliicoccaceae Methanomassiliicoccus |
| Acidobacteria Holophagae Subgroup_7 NA NA |
| Actinobacteria Actinobacteria Frankiales Geodermatophilaceae Modestobacter |
| Actinobacteria Coriobacteriia OPB41 NA NA |
| Actinobacteria Thermoleophilia Gaiellales NA NA |
| Armatimonadetes DG-56 NA NA NA |
| Bacteroidetes Bacteroidia Bacteroidales Prolixibacteraceae BSV13 |
| Bacteroidetes Bacteroidia Bacteroidales SB-5 NA |
| Bacteroidetes Ignavibacteria Kryptoniales BSV26 NA |
| Bacteroidetes Ignavibacteria OPB56 NA NA |
| Chloroflexi KD4-96 NA NA NA |
| Chloroflexi Ktedonobacteria C0119 NA NA |
| Chloroflexi Ktedonobacteria Ktedonobacterales JG30-KF-AS9 NA |
| Chloroflexi Ktedonobacteria Ktedonobacterales Ktedonobacteraceae 1921-2 |
| Cyanobacteria Melainabacteria Gastranaerophilales NA NA |
| Firmicutes Bacilli Bacillales Alicyclobacillaceae Tumebacillus |
| Firmicutes Bacilli Bacillales NA NA |
| Firmicutes Clostridia Clostridiales Clostridiaceae_1 Fonticella |
| Firmicutes Clostridia Clostridiales Clostridiaceae_1 NA |
| Firmicutes Clostridia Clostridiales Heliobacteriaceae Hydrogenispora |
| Firmicutes Clostridia Clostridiales NA NA |
| Firmicutes Clostridia Clostridiales Ruminococcaceae NA |
| Gemmatimonadetes Gemmatimonadetes Gemmatimonadales Gemmatimonadaceae NA |
| Patescibacteria Saccharimonadia Saccharimonadales Saccharimonadaceae NA |
| Planctomycetes Planctomycetacia Pirellulales Pirellulaceae Pir4_lineage |
| Planctomycetes Planctomycetacia Planctomycetales Gimesiaceae NA |
| Proteobacteria Alphaproteobacteria Rhizobiales Xanthobacteraceae Pseudolabrys |
| Proteobacteria Alphaproteobacteria Rhodospirillales Magnetospirillaceae NA |
| Proteobacteria Deltaproteobacteria Myxococcales Polyangiaceae NA |
| Proteobacteria Deltaproteobacteria NA NA NA |
| Proteobacteria Gammaproteobacteria Betaproteobacteriales SC-I-84 NA |
| Rokubacteria NC10 Methylomirabilales Methylomirabilaceae Sh765B-TzT-35 |

| **Unique to the natural anoxic and natural oxic-anoxic** **active communities** |
| --- |
| Crenarchaeota Bathyarchaeia NA NA NA |
| Acidobacteria Acidobacteriia Subgroup_12 NA NA |
| Bacteroidetes Bacteroidia Sphingobacteriales AKYH767 NA |
| Chlamydiae Chlamydiae Chlamydiales cvE6 NA |
| Proteobacteria Gammaproteobacteria JG36-TzT-191 NA NA |
| Verrucomicrobia Verrucomicrobiae Chthoniobacterales Xiphinematobacteraceae Candidatus_Xiphinematobacter |

| **Unique to the natural anoxic and natural oxic** **active communities** |
| --- |
| Bacteroidetes Bacteroidia Chitinophagales 37-13 NA |
| Bacteroidetes Bacteroidia Sphingobacteriales KD1-131 NA |
| Chloroflexi Dehalococcoidia NA NA NA |
| Firmicutes Clostridia Clostridiales Clostridiaceae_1 Clostridium_sensu_stricto_12 |
| Planctomycetes Phycisphaerae Tepidisphaerales NA NA |
| Proteobacteria Alphaproteobacteria Acetobacterales Acetobacteraceae Acidocella |
| Proteobacteria Deltaproteobacteria Syntrophobacterales Syntrophaceae Desulfobacca |
| Proteobacteria Deltaproteobacteria Syntrophobacterales Syntrophaceae Desulfomonile |
| Proteobacteria Deltaproteobacteria Syntrophobacterales Syntrophobacteraceae Desulfovirga |

| **Unique to the restored oxic** **and natural oxic** **active communities** |
| --- |
| Actinobacteria Actinobacteria Micrococcales Micrococcaceae Rothia |
| Bacteroidetes Bacteroidia Chitinophagales NA NA |
| Kiritimatiellaeota Kiritimatiellae WCHB1-41 NA NA |
| Planctomycetes Planctomycetacia Isosphaerales Isosphaeraceae Candidatus_Nostocoida |

| **Unique to the natural anoxic and restored anoxic active communities** |
| --- |
| Acidobacteria Acidobacteriia Acidobacteriales Acidobacteriaceae_(Subgroup_1) Granulicella |
| Acidobacteria Acidobacteriia Acidobacteriales Acidobacteriaceae_(Subgroup_1) Terracidiphilus |
| Actinobacteria Actinobacteria Corynebacteriales Mycobacteriaceae Mycobacterium |
| Bacteroidetes Ignavibacteria Ignavibacteriales NA NA |
| Chlamydiae Chlamydiae Chlamydiales NA NA |
| Dependentiae Babeliae Babeliales Babeliaceae NA |
| Dependentiae Babeliae Babeliales Vermiphilaceae NA |
| Planctomycetes Planctomycetacia Isosphaerales Isosphaeraceae Isosphaera |
| Proteobacteria Alphaproteobacteria Rhizobiales Beijerinckiaceae Methylocystis |
| Proteobacteria Alphaproteobacteria Rhodospirillales NA NA |
| Proteobacteria Deltaproteobacteria Oligoflexales Oligoflexaceae NA |

| **Found in all active communities** |
| --- |
| Thaumarchaeota Group_1.1c NA NA NA |
| Acidobacteria Acidobacteriia Acidobacteriales NA NA |
| Acidobacteria Acidobacteriia Subgroup_13 NA NA |
| Acidobacteria Acidobacteriia Subgroup_2 NA NA |
| Acidobacteria Acidobacteriia Solibacterales Solibacteraceae_(Subgroup_3) Bryobacter |
| Acidobacteria Acidobacteriia Solibacterales Solibacteraceae_(Subgroup_3) Candidatus_Solibacter |
| Acidobacteria Subgroup_6 NA NA NA |
| Actinobacteria Acidimicrobiia IMCC26256 NA NA |
| Actinobacteria Acidimicrobiia NA NA NA |
| Chloroflexi Ktedonobacteria Ktedonobacterales Ktedonobacteraceae NA |
| Patescibacteria Saccharimonadia Saccharimonadales NA NA |
| Planctomycetes Phycisphaerae Phycisphaerales AKAU3564_sediment_group NA |
| Planctomycetes Phycisphaerae Tepidisphaerales WD2101_soil_group NA |
| Planctomycetes Planctomycetacia Gemmatales Gemmataceae NA |
| Planctomycetes Planctomycetacia Pirellulales Pirellulaceae NA |
| Proteobacteria Alphaproteobacteria Elsterales NA NA |
| Proteobacteria Deltaproteobacteria Myxococcales Polyangiaceae Pajaroellobacter |
| Proteobacteria Gammaproteobacteria Gammaproteobacteria_Incertae_Sedis Unknown_Family Acidibacter |
| Verrucomicrobia Verrucomicrobiae Pedosphaerales Pedosphaeraceae NA |

# Supplemental Figures


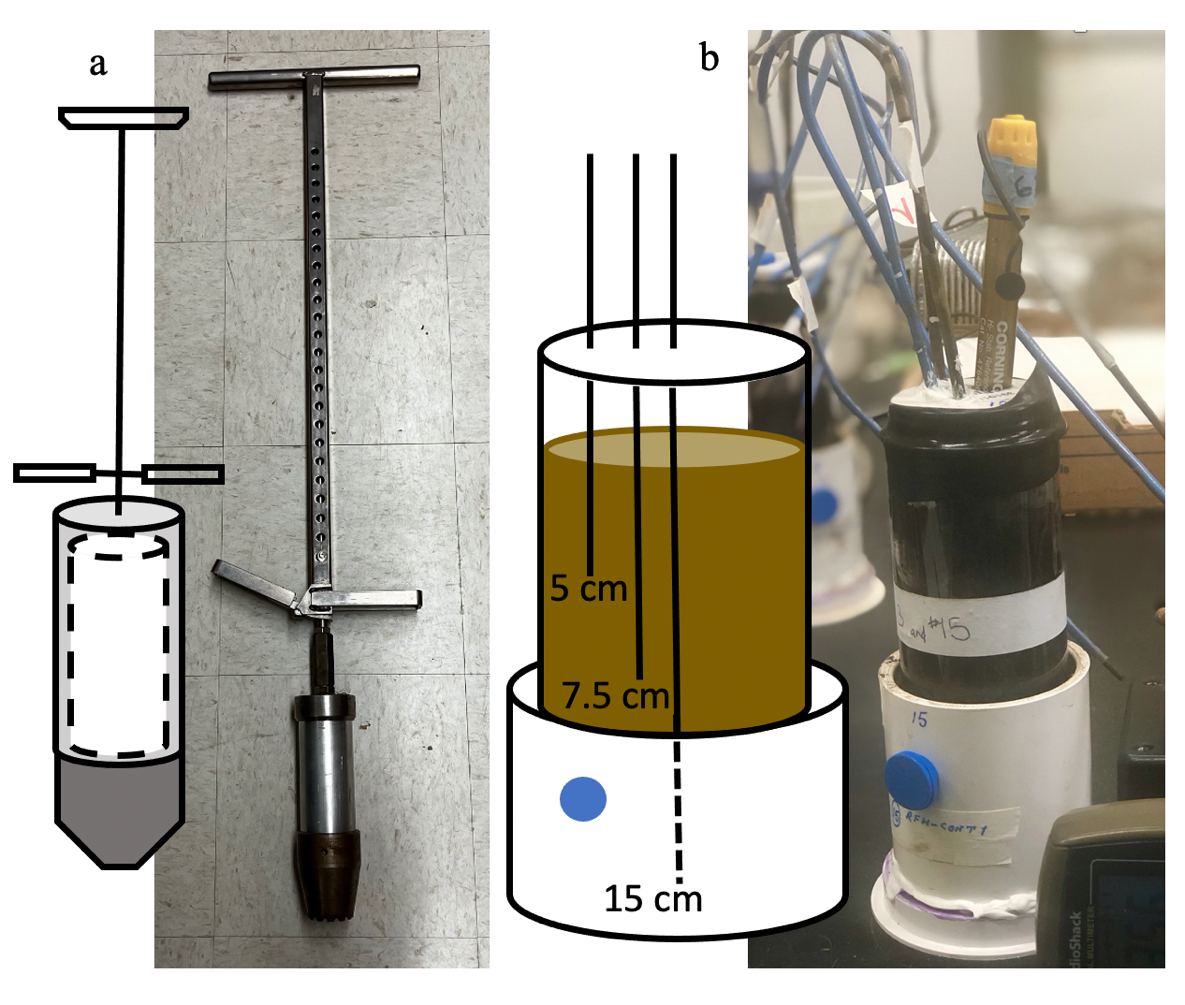


## Figure S1. Diagram of incubation design. a.) Soil cores were collected from wetlands in intact glass sleeves using custom designed soil corer. Soil cores were 15 cm deep and 2.54 cm in diameter. b.) Glass sleeves measured 20 cm, leaving 5 cm of headspace above the 15 cm soil core. Cores were sealed with air-tight caps. Redox probes were installed at 5 cm, 7.5 cm, and 15 cm depth in the restored cores and 5 cm and 10 cm depth in the natural cores. A septum at the bottom of the core stand allowed saturation to be maintained. Soil drainage occurred out of the bottom of the core.


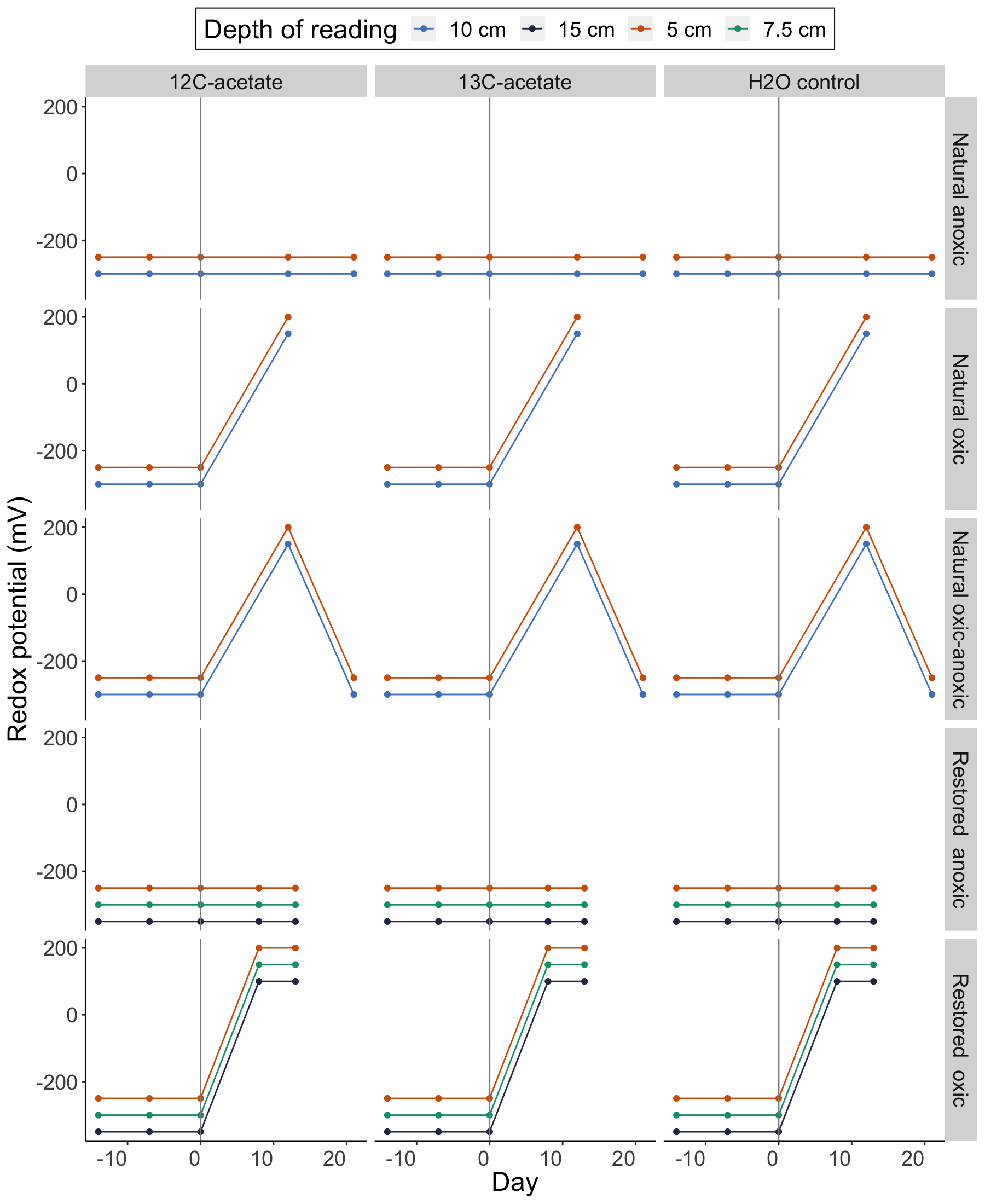


## Figure S2. Schematic represents anticipated change in redox (mV) at varying depths for each of the redox condition groups (y-axis facet) and the acetate additions (x-axis facet). Within each group and acetate addition type there are three replicate cores. Depth of redox reading within the core is indicated by color. The anticipated first redox measurements are from the pre-incubation (day -14 and -7), followed by a measurement made at the initiation (day 0) of the incubations. Vertical line at day 0 indicates beginning of incubation.


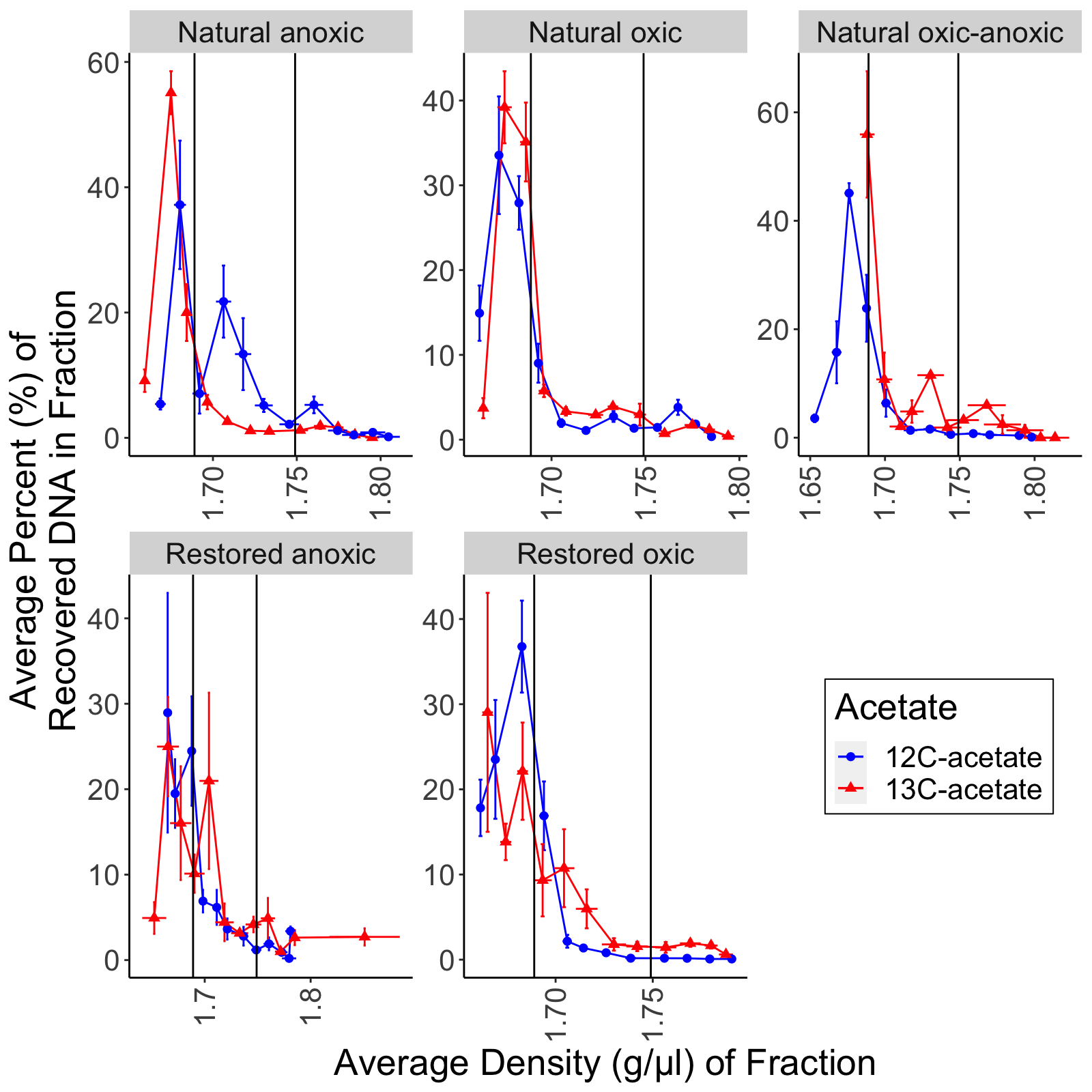


## Figure S3. Average percent (%) of total recovered DNA in each of the 12 fractions plotted by the average density (g/µL) of corresponding fraction. Fractions to the left of the vertical black line at 1.69 g/µL indicates light density fractions. Fractions to the left of vertical black line at 1.75 g/µL indicate medium density fractions. Fractions to the right of vertical black line at 1.75 g/µL indicate the heavy density fraction. Vertical error bars indicate the standard error of mean for % DNA in fractions. Horizontal error bars indicate the standard error of mean for density of each fraction.


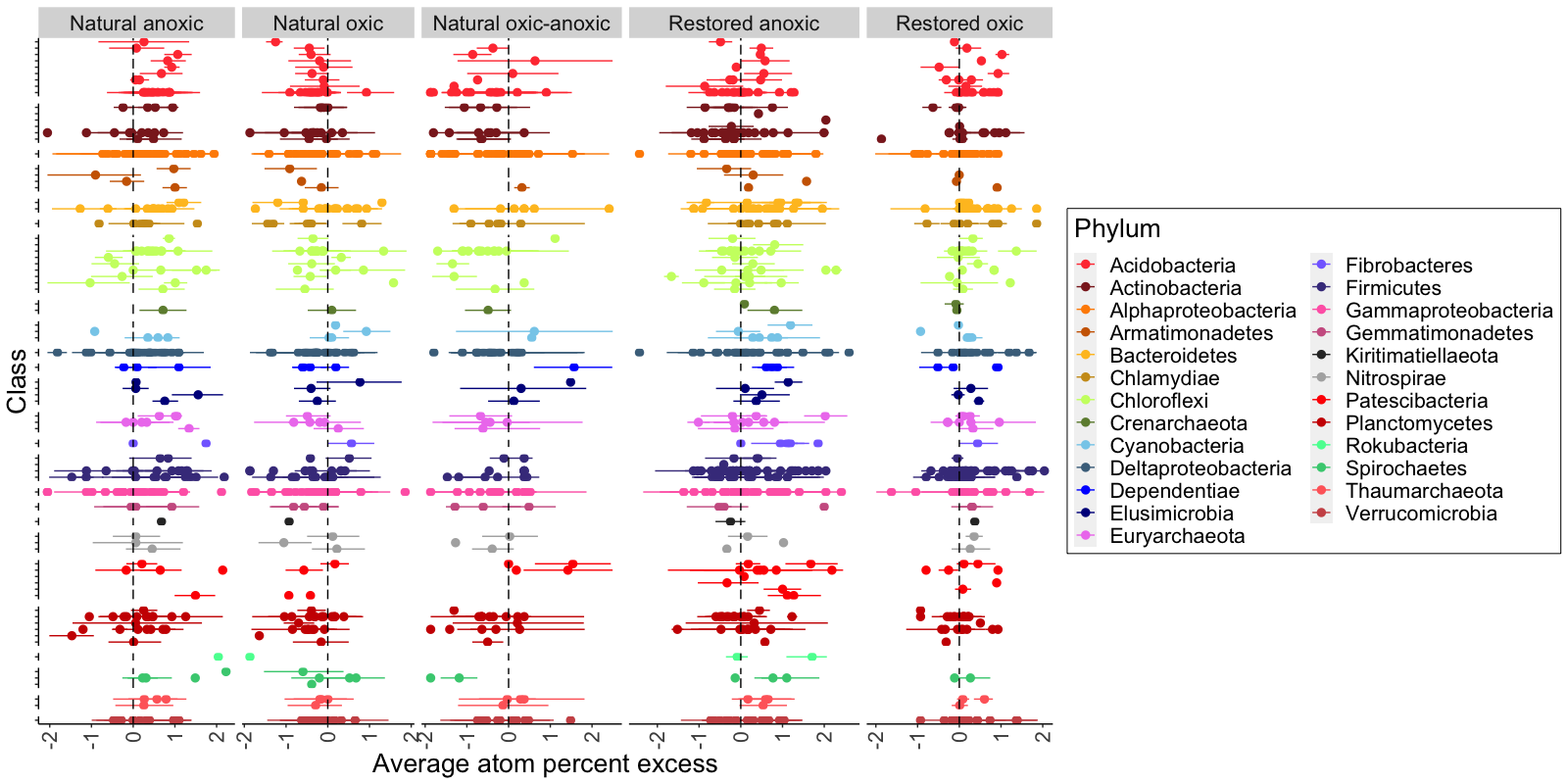


## Figure S4. Average atom percent excess (APE) of ^13^C in DNA in each class, grouped by phyla and characterized by wetland type and redox condition. Positive APE represents ^13^C incorporation into the DNA. Each dot represents the average APE of a class within the phylum. Horizontal bars show average bootstrapped 95% confidence intervals around each class’s mean APE. Phyla are color coded. Note independent x and y axis scale


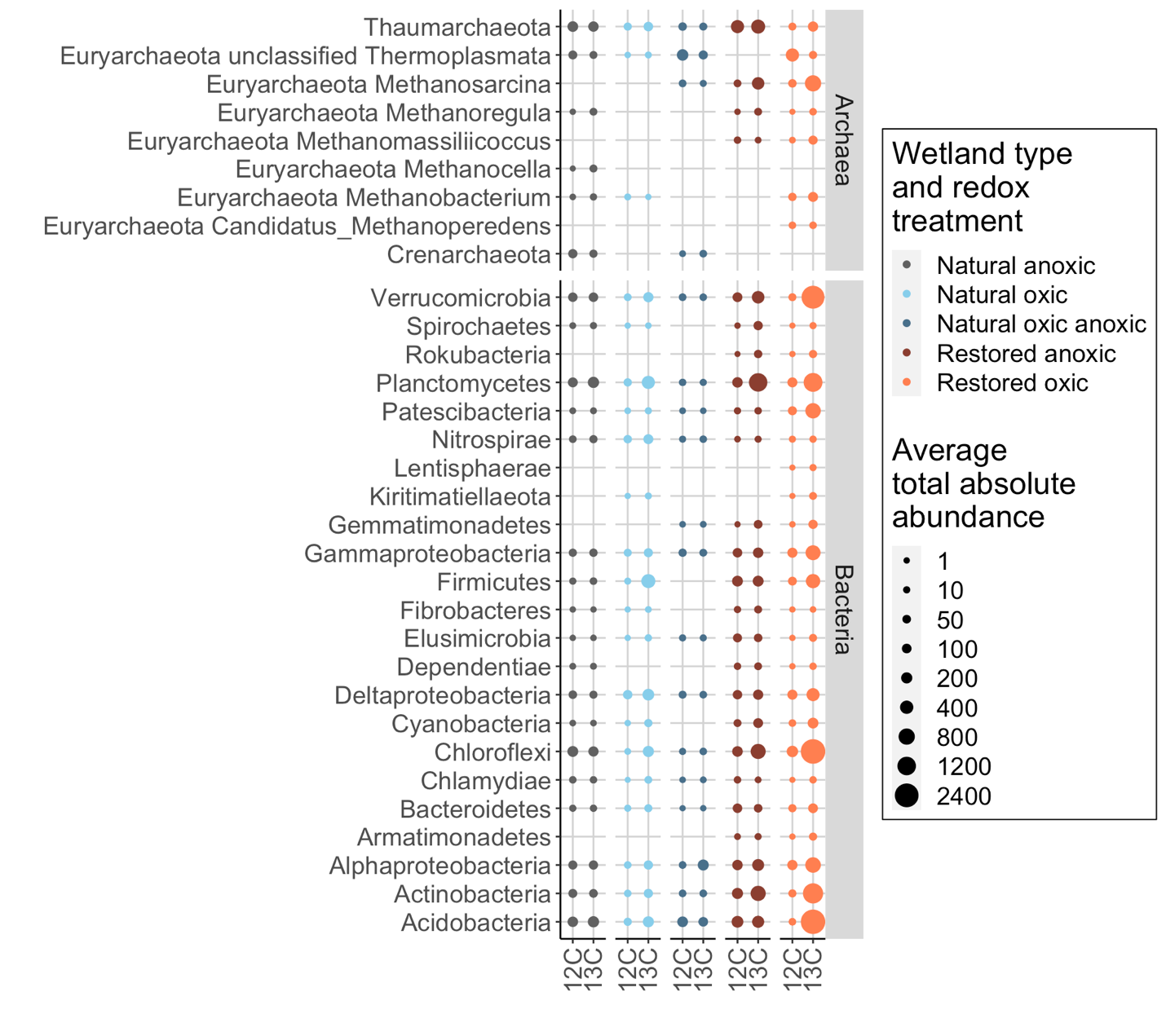


## Figure S5. Average total absolute abundance (relative abundance normalized by gene copies/ng of DNA) of all active taxa in both the ^12^C and ^13^C labeled cores (x-axis). Taxa are grouped by phylum and by genera for methanogens in the phylum *Euryarchaeota*. Figure is sorted alphabetically by phylum and facetted by kingdom (Archaea and Bacteria) as well as redox treatment. Size of the circle corresponds to average total absolute abundance in the wetland’s different redox treatment communities. Color indicates wetland type and redox treatment. Treatments are listed left to right: natural oxic, natural oxic-anoxic, natural anoxic, restored oxic, and restored anoxic.


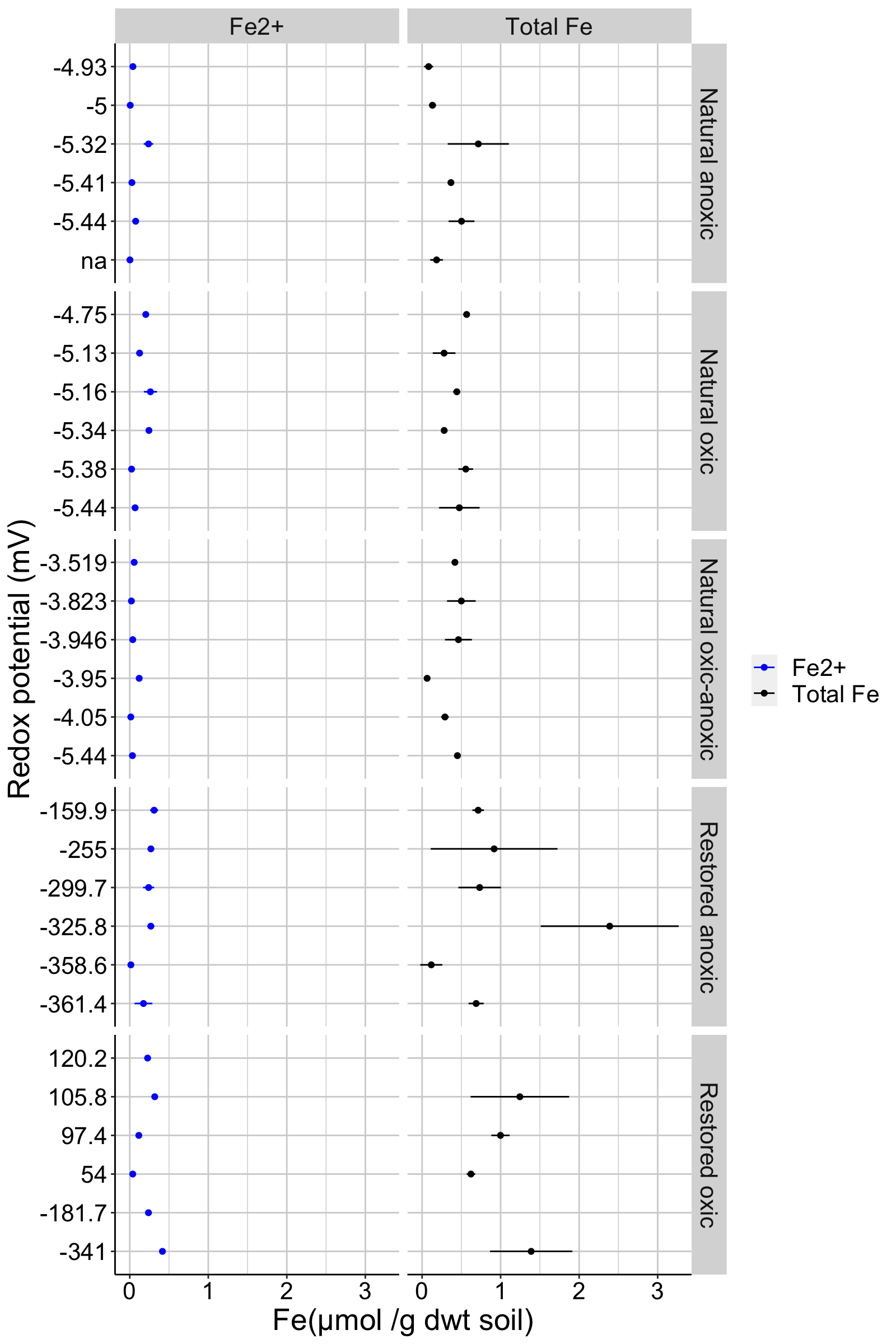


## Figure S6. Average concentration of Fe forms in each core plotted according to corresponding final redox (mV) measurement in the core. The left panel plots average Fe^2+^ concentration (blue) in each core and right panel plots average total Fe concentration (black) in each core. Horizontal bars correspond to standard deviation. Total Fe and Fe^2+^ concentrations positively corelate (linear regression, p = 0.002, R^2^ = 0.29). Average Fe^2+^ concentrations were higher in the restored wetland (ANOVA, p = 0.004), as were average concentrations of total Fe (ANOVA, p = 0.007). Note independent y axis scale. NA indicates no measurement taken.


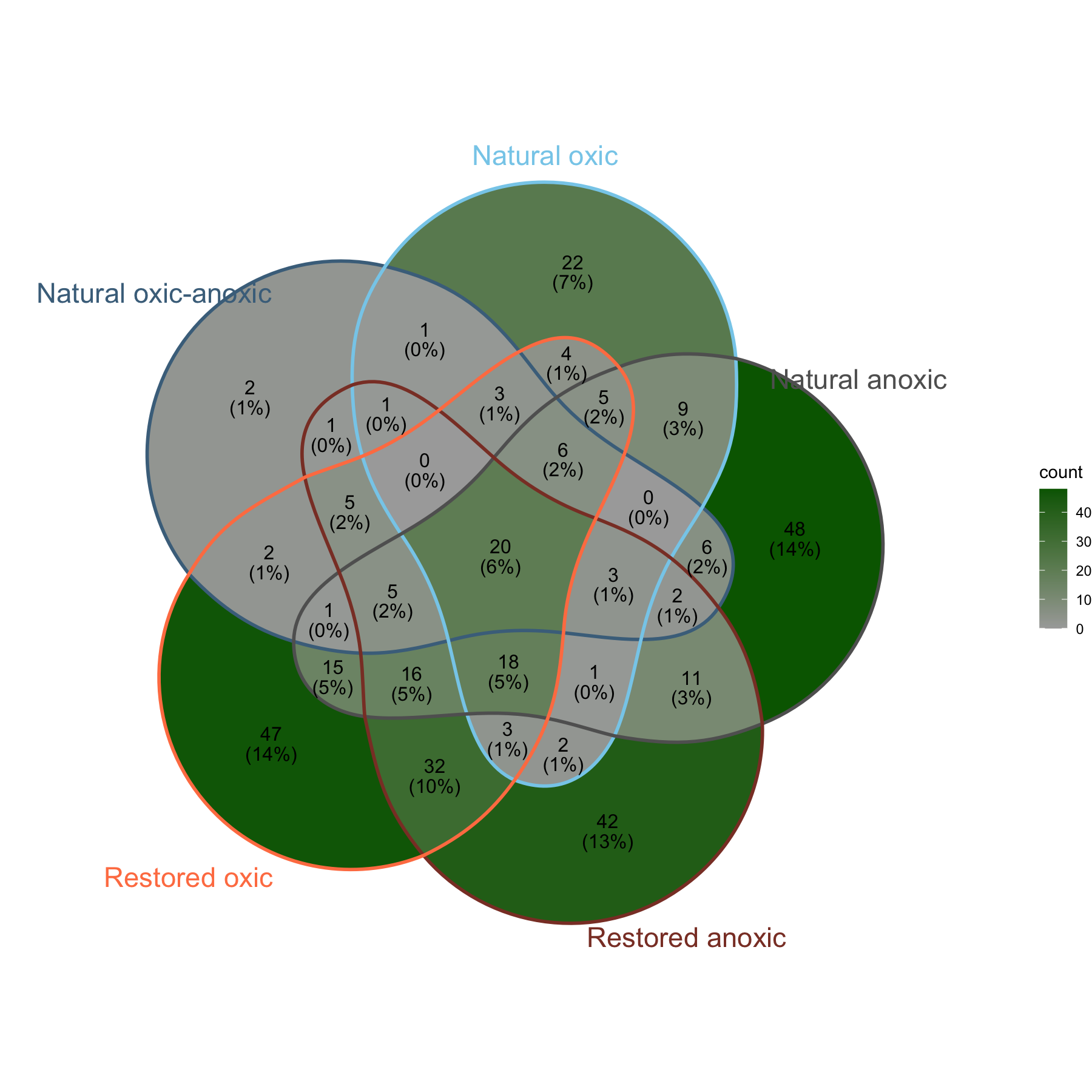


## Figure S7. Venn Diagram showing the number of genera that co-occur, or are independent, in the wetland redox conditions. Outline color represents the redox condition. The background color represents the number of taxa in that section. A higher count of genera is indicated by a deeper green background, while a lower count is indicated by a grayer background. The number in each section is the number of genera, percent total genera is shown below (x%).
